# WASP family proteins and formins compete in pseudopod- and bleb-based migration

**DOI:** 10.1083/jcb.201705160

**Published:** 2018-02-05

**Authors:** Andrew J. Davidson, Clelia Amato, Peter A. Thomason, Robert H. Insall

**Affiliations:** Cancer Research UK Beatson Institute, Glasgow, Scotland, UK

## Abstract

Actin pseudopods induced by SCAR/WAVE drive normal migration and chemotaxis in eukaryotic cells. Cells can also migrate using blebs, in which the edge is driven forward by hydrostatic pressure instead of actin. In *Dictyostelium discoideum*, loss of SCAR is compensated by WASP moving to the leading edge to generate morphologically normal pseudopods. Here we use an inducible double knockout to show that cells lacking both SCAR and WASP are unable to grow, make pseudopods or, unexpectedly, migrate using blebs. Remarkably, amounts and dynamics of actin polymerization are normal. Pseudopods are replaced in double SCAR/WASP mutants by aberrant filopods, induced by the formin dDia2. Further disruption of the gene for dDia2 restores cells’ ability to initiate blebs and thus migrate, though pseudopods are still lost. Triple knockout cells still contain near-normal F-actin levels. This work shows that SCAR, WASP, and dDia2 compete for actin. Loss of SCAR and WASP causes excessive dDia2 activity, maintaining F-actin levels but blocking pseudopod and bleb formation and migration.

## Abstract (precis)

In migrating cells, actin underlies formation of pseudopods, filopods, and blebs. Davidson et al. use multiple knockouts in *Dictyostelium* to show that WASP family proteins SCAR/WAVE and WASP compete with the formin dDia2 for actin, influencing pseudopod and bleb formation.

## Introduction

The actin cytoskeleton is involved in almost all aspects of cell behavior, but most clearly cell migration, endocytosis, adhesion, and cell division (Insall and Machesky, 2009). Each depends on formation of multiple actin-based structures through the coordinated activity of various actin regulatory proteins. How different combinations of these proteins are brought together at the right time and place within the cell to promote the formation of a particular actin-based structure is still poorly understood. Cell migration is further complicated by the fact that cells can use a wide repertoire of actin regulators to form several different types of protrusion with different dynamics.

The Arp2/3 complex generates thick meshes of branched F-actin (Pollard, 2007) that support the formation of broad cellular protrusions known as pseudopods or lamellipods. Conversely, formins form unbranched actin filaments (Pollard, 2007), which are used in several different cellular processes. Filopods—narrow cellular projections made of bundled actin filaments—may be made from formin- or Arp2/3-generated actin. Cells can also extend their edges using blebs, which form when actin fills an area of plasma membrane that has detached and bulged outward from the cell (Paluch and Raz, 2013). Neither the Arp2/3 complex nor formins are enriched at sites where blebs occur, although the actin that polymerizes behind blebs after they have formed contains some Arp2/3 complex (Tyson et al., 2014). Contractility driven by actin and the motor protein myosin II (collectively actomyosin) is essential for the initial separation of the plasma membrane from the underlying actin cortex (Diz-Muñoz et al., 2010), but the regulators that catalyze the actin polymerization observed as blebs are filled are unknown.

Pseudopods, filopods, and blebs can be used individually or in different combinations to promote cell motility. This is perhaps most evident in the highly motile *Dictyostelium discoideum*, which is widely used for studies of actin regulation and cell migration. *Dictyostelium* normally migrates by extending a mixture of both pseudopods and blebs (Tyson et al., 2014). However, cell migration can be driven to favor pseudopods or blebs using genetics or pharmacology or by physical means. For instance, inhibition of actomyosin contractility inhibits blebbing (Yoshida and Soldati, 2006; Ibo et al., 2016), whereas subjecting cells to increased compression favors blebbing over pseudopod formation (Zatulovskiy and Kay, 2016).

In all eukaryotic cells, the subcellular localization and activity of the Arp2/3 complex are controlled by members of the WASP family (Derivery and Gautreau, 2010). Mammalian WASP nomenclature is confusing: WASP itself (named after the gene mutated in Wiskott-Aldrich syndrome) is restricted to blood cells and has an unusual role, whereas N-WASP (originally, but incorrectly named neural WASP), is ubiquitously expressed. Other members of the WASP family include SCAR/WAVE and WASH (Derivery and Gautreau, 2010). *Dictyostelium* possesses a single, well-conserved member of each of the WASP (Myers et al., 2005), SCAR (Bear et al., 1998), and WASH (Carnell et al., 2011) families. This simplicity makes it an ideal organism to separate and understand the roles of WASP, SCAR/WAVE, and WASH.

Like WASPs from other organisms, *Dictyostelium* WASP colocalizes with clathrin-coated pits (CCPs), coinciding with actin-driven vesicle internalization (Veltman and Insall, 2010). Its localization contrasts with that of SCAR, which is normally found at the tips of growing pseudopods during migration. A study from several years ago asserted a fundamental role for WASP in pseudopod extension and cell viability (Myers et al., 2005), but there has been little supporting evidence for this view. We recently found that WASP is able to substitute for SCAR and appears to be responsible for the residual pseudopods extended by *Dictyostelium scar* knockout cells (Veltman et al., 2012); this was unexpected as the two are typically thought to be regulated by different upstream pathways, but has since been confirmed in *Caenorhabditis elegans* (Zhu et al., 2016). Despite the remarkable ability of *Dictyostelium* WASP to change its behavior to compensate for the loss of SCAR, it is not sufficient to maintain a normal rate of pseudopod formation, and migrating cells without *scar* make blebs at an increased rate. Therefore, cell motility is maintained in *scar*-null *Dictyostelium* through a combination of WASP-driven pseudopods and Arp2/3 complex–independent blebbing (Veltman et al., 2012).

Whether SCAR and, in the absence SCAR, WASP are the only proteins capable of promoting pseudopod extension is so far unknown. Furthermore, it is not understood how blebbing is regulated, nor what makes blebbing increase in the absence of SCAR, though it clearly maintains efficient motility. Current signal-based models of motility suggest that the formation of different protrusions is achieved solely by different upstream signals—for example, Rac1 specifically activating the Arp2/3 complex to extend a pseudopod, and RhoA/B/C regulating Diaphanous-related formins to create a filopod. The existence of cross talk between these pathways is accepted, but it is frequently presumed that any given protrusion is initiated by a single upstream pathway. The ability to switch between pseudopod- and bleb-based motility in the short term requires a degree of cytoskeletal plasticity that is not explained by such models.

Recently, competition between different actin regulators for actin monomers has been shown to influence the form of an actin-based structure that is assembled (Burke et al., 2014; Rotty and Bear, 2014; Lomakin et al., 2015). The possibility of competition between regulators remains to be fully explored. However, it offers an attractive explanation for how the activity of a diverse set of actin regulators can be integrated and rapidly modulated to help drive dynamic behavior such as cell motility (Davidson and Wood, 2016).

Here we describe a *Dictyostelium* mutant lacking WASP and show that it is unexpectedly both viable and able to make normal pseudopods. However, when SCAR/WAVE is also lost, pseudopods are entirely abolished, demonstrating that only WASP can substitute for SCAR during pseudopod-based migration. Surprisingly, cells deficient in both SCAR and WASP are unable to switch to bleb-based motility, rendering them essentially immobile. These cells instead form an excessive number of filopods, with the Diaphanous-related formin dDia2 (Junemann et al., 2016) at their tips. When filopod formation was suppressed by the additional mutation of *ddia*2, bleb-based motility was restored. Our data therefore show that the activity of dDia2 is counterbalanced by that of the Arp2/3 complex. In the absence of Arp2/3 complex, unconstrained dDia2 activity overwhelms the cytoskeleton and blocks cell movement. Based on these findings, we concluded that *Dictyostelium* cell migration is a product of a dynamic competition between different actin regulators. We also propose that competition is a general principle underlying regulation of the actin cytoskeleton.

## Results

### WASP is not required for *Dictyostelium* cell growth or chemotaxis

An earlier study (Myers et al., 2005) concluded that *Dictyostelium* WASP’s principal role was in pseudopod generation and maintenance, and was therefore essential for cell viability. However, this now seems surprising as mammalian cells survive comfortably without N-WASP, and *Dictyostelium* WASP is found in clathrin pits, not normally at pseudopods (Veltman et al., 2012). We therefore tested WASP’s roles with no threat of lethality or genetic suppression by generating a WASP-inducible knockout (WIKO) in which the genomic copy of WASP is fully disrupted and replaced by an expression construct in which GFP-tagged WASP is controlled by a *tet* promoter (Fig. S1 A). In these cells, the expression of WASP depends on doxycycline (DOX) in the medium; no WASP at all is detectable without it (Fig. S1 B). Surprisingly, WIKO cells grew normally when WASP production was completely suppressed, showing that WASP is in fact dispensable for cell viability. We confirmed this observation by disrupting the gene for WASP, *was*A, in a normal axenic background to create a simple knockout (*was*A^−^; Fig. S1 B). Again, the loss of *was*A had no adverse affect on cell growth in liquid medium (Fig. S1 C), though the mutant cells grew very poorly on bacteria (Fig. S1 D), suggesting that phagocytic uptake is more seriously affected than macropinocytosis. Mutant cells also usually failed to aggregate (Fig. S1 E) or express the cAMP receptor (Fig. S1 F) when starved, though the completeness of this phenotype was variable. We have therefore used folate, rather than cAMP, as a chemoattractant in chemotaxis and motility assays.

**Figure 1.**
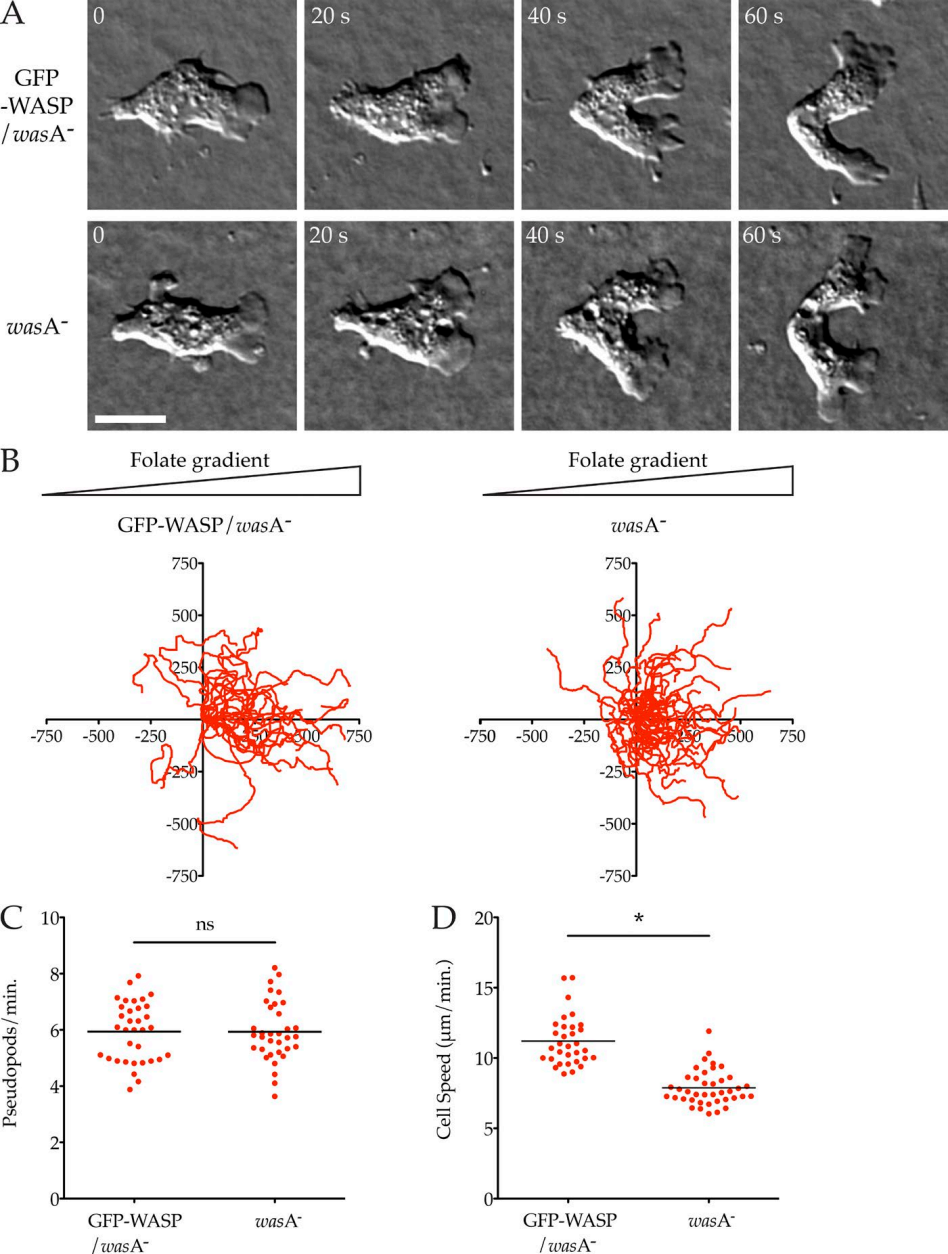
**WASP is not required for chemotaxis or pseudopod formation. (A)** Normal pseudopods in WASP knockout cells. *was*A^−^ cells ± GFP-WASP were allowed to chemotax to folate under agarose and examined by DIC microscopy. A representative cell is shown. See also Video 1. Cells are shown at 20-s intervals. Bar, 5 µm. **(B)** Robust chemotaxis in WASP knockout cells. Cells (as in A) were tracked showing equivalent, strong bias in the direction of the chemoattractant (>20 cells/line from three independent assays; triangles indicate direction of the folate gradient; scale is in micrometers). **(C)** Rate of pseudopod formation. GFP-WASP/*was*A^−^ control and the *was*A^−^ mutant showed identical rates of pseudopod formation (5.94 vs. 5.93 pseudopods/min, respectively). **(D)** Diminished speed in WASP knockout cells. Speeds were derived from tracks in B, showing a decrease in wasA^−^ cells compared with the GFP-WASP/*was*A^−^ control knockout (7.88 ± 0.20 vs. 11.20 ± 0.31 µm/min, mean ± SEM; P < 0.0001, unpaired Student’s *t* test).

We found *was*A^−^ cells extended morphologically normal pseudopods, of normal sizes, and migrated robustly (Fig. 1, A and B; and Video 1). Quantitative assessment of pseudopod dynamics while cells chemotaxed under agar (Blagg et al., 2003) toward folate showed that knockout cells form pseudopods at a normal rate (Fig. 1 C; *was*A^−^ mutant 5.93 ± 0.18 pseudopods/min, GFP-WASP/*was*A^−^ controls 5.94 ± 0.18 pseudopods/min, mean ± SEM). Detailed analysis of the cell tracks revealed a slight but statistically significant ∼30% decrease in cell speed (Fig. 1 D, P < 0.0001) in *was*A^−^ cells, implying that WASP does not contribute to pseudopods in normal cells, but does affect migration through a different mechanism.

**Figure 2.**
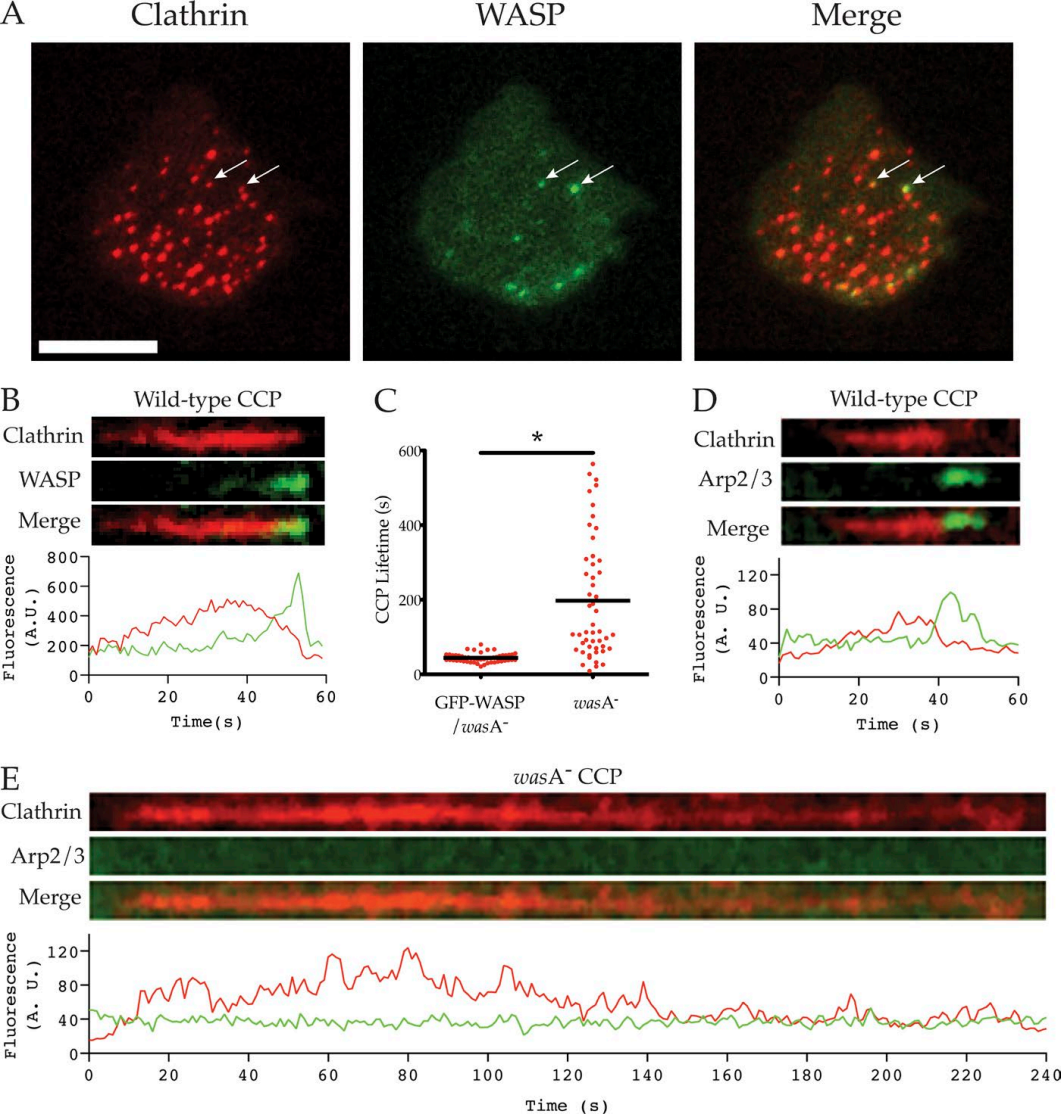
**Defects in clathrin-mediated endocytosis. (A)** Colocalization of WASP and clathrin pits. *was*A^−^ cells were transfected with CLC-mRFP (clathrin) and GFP-WASP (giving a wild-type WASP phenotype) and imaged by TIRF. WASP colocalizes with a subset of clathrin pits at any time (white arrows). Bar, 10 µm. **(B)** Colocalization of clathrin and WASP at puncta. Wild-type cells were transfected with CLC-mRFP (clathrin) and GFP-WASP, and clathrin-mediated endocytosis was visualized using TIRF microscopy. Kymograph and fluorescence intensity plot demonstrate the dynamic localization of clathrin and WASP at clathrin pits (representative of >50 puncta from >20 cells). **(C)** Vesicle internalization during clathrin-mediated endocytosis. *was*A^−^ cells were transfected with CLC-mRFP alone or CLC-mRFP and GFP-WASP and visualized by TIRF. Clathrin punctum lifetime (time between appearance and disappearance from TIRF field of view) was measured from 50 pits/cell line over two independent experiments. Clathrin puncta in WASP knockout cells were far longer lived than in GFP-WASP/*was*A^−^ controls (197.5 ± 22.8 vs. 44.2 ± 1.7 s, mean ± SEM); P < 0.0001, unpaired Student’s *t* test). (D and E) Recruitment of Arp2/3 complex. Cells were transfected with CLC-mRFP (clathrin) and GFP-ArpC4 (Arp2/3 complex), and clathrin-mediated endocytosis was visualized using TIRF microscopy. Kymographs and accompanying fluorescence intensity plots demonstrate the dynamic localization of clathrin and the Arp2/3 complex at clathrin pits (representative of >50 puncta from >20 cells). **(D)** In wild-type cells, recruitment of the Arp2/3 complex to clathrin pits coincides with internalization. **(E)** In the *was*A^−^ mutant, many clathrin pits fail to recruit the Arp2/3 complex and persist on the plasma membrane for hundreds of seconds.

Although *was*A mutant cells had normal pseudopods, they struggled to retract their trailing tails efficiently, explaining their reduced migratory speed (Video 2). Given N-WASP’s established roles in clathrin-mediated endocytosis (Merrifield et al., 2004), and because *Dictyostelium* clathrin (*chc*A) mutant cells also have uropod defects (Damer and O’Halloran, 2000; Wessels et al., 2000), we explored CCP uptake in *was*A^−^ cells. When visualized by total internal reflection fluorescence (TIRF) microscopy, GFP-WASP colocalizes with a subset of mRFP-tagged clathrin light chain (CLC-mRFP; Fig. 2 A) puncta. Clathrin-mediated endocytosis is a brief and tightly regulated event in control cells, lasting 44 ± 2 (mean ± SEM) seconds in our hands, similar to the previous result of 39 s (Brady et al., 2010). As shown by kymographs and intensity plots (Fig. 2 B), clathrin in puncta accumulates over tens of seconds until a short (3-4-s) burst of WASP is recruited (Video 3), which induces CCP internalization and loss from TIRF images. Thus at any given time only a small proportion of coated pits have associated WASP, but nearly all pits eventually recruit WASP.

The clathrin puncta of *was*A^−^ cells were consistently trapped on the basal membrane for fivefold longer (198 ± 23 s; Fig. 2 C) than in controls. Even this is an underestimate, as many of the CCPs in the *was*A^−^ mutant had a lifetime greater than the length of the time-lapse videos. To observe recruitment of the Arp2/3 complex to CCPs, we coexpressed CLC-mRFP and GFP-ArpC4. Just as with WASP, the Arp2/3 complex is normally recruited immediately before CCP internalization (Fig. 2 D). In contrast, many clathrin puncta in the *was*A^−^ cells persisted on the plasma membrane for hundreds of seconds, with no discernible recruitment of the Arp2/3 complex (Fig. 2 E).

Additional similarities between WASP and clathrin mutants included a growth defect when introduced to shaking culture (Fig. S2 A). As with c*hc*A^−^ mutants (Wessels et al., 2000), *was*A^−^ mutant cells became multinucleate when grown in suspension (Fig. S2, B and C). Although *was*A^−^ cells grow normally when provided with a substrate to adhere to (Fig. S1 B), they exhibited cleavage furrow abnormalities and took longer to divide than wild-type cells (Fig. S2 D). This is a “classic” phenotype, epitomized by the myosin II (*mhc*A) mutant (De Lozanne and Spudich, 1987; Knecht and Loomis, 1987), whereby mitotic cells can overcome cytokinesis defects by pulling themselves apart in an adhesion-dependent manner. As has been shown for the *chc*A mutant, *was*A^−^ cells fail to robustly recruit myosin II to the cleavage furrow during cell division (Fig. S2 E). Similarly, the localization of myosin II to the trailing tail of chemotaxing *was*A^−^ cells was also perturbed (Video 4 and Fig. S2 F).

We conclude that WASP’s principal role is to promote clathrin-mediated endocytosis, and it is not normally required for pseudopod formation. The tail retraction defect, which subtly impairs efficient cell migration, appears more related to endocytosis than pseudopod dynamics.

### Generation of a double *scar*/*wasp* mutant

**Figure 3.**
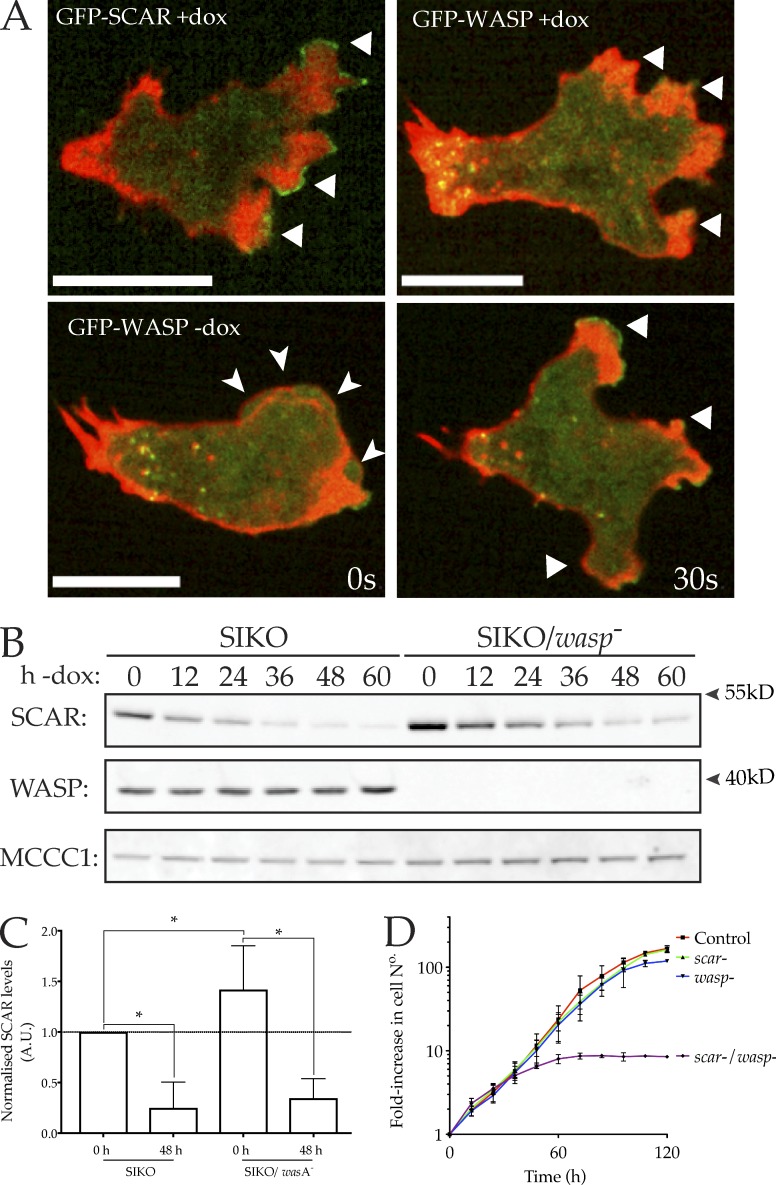
**Generation of a double *scar/wasp* mutant. (A)** SCAR and WASP localize as expected in SIKO cells. Top: GFP-SCAR localizes to pseudopods (arrows), whereas GFP-WASP is not found at leading edges in the presence of SCAR, and is restricted to puncta at the center and rear. Bottom: When SCAR is repressed, GFP-WASP relocalizes to pseudopods but not blebs (arrowheads; green: GFP-SCAR and GFP-WASP; red: RFP-LifeAct). Bar, 5 µm. **(B and C)** Loss of both SCAR and WASP from SIKO/*wasp^−^* cells verified by Western blotting with anti-SCAR and anti-WASP antibodies. In each case, substantial repression of SCAR is seen between 48 and 60 h. SIKO/*wasp^−^* cells are completely deleted for WASP; only SCAR is inducible. MCCC1 is the loading control (Davidson et al., 2013a). **(C)** Quantitation of several repeats normalized to the corresponding MCCC1 band, then to SIKO + DOX scar levels (*n* = 3 blots, one-way ANOVA, difference between SIKO and SIKO/*wasA*^−^ at 48 h not significant, P = 0.7135). **(D)** Loss of both SCAR and WASP causes growth arrest. Cells were grown in plates in axenic medium and counted every 12 h. SIKO/*wasp*^−^ cells were starved of DOX at time = 0 h. Around 60 h later, when SCAR expression is lost, growth completely ceases in SIKO/*wasp^−^* cells but not controls lacking either *scar* or *wasp* alone (*n* = 3).

WASP is not necessary for pseudopods in normal cells, but it becomes localized to pseudopods in *scar* mutants (Veltman et al., 2012). We therefore tested whether WASP is truly responsible for the remaining protrusions of *scar^−^* cells, and to determine what other proteins (WASP family or otherwise) can take on SCAR’s role in pseudopods. To achieve this, we set about creating cells devoid of both SCAR and WASP. We were unable to knock out WASP in a *scar*-null background, implying the combination is lethal. We therefore adopted an inducible approach, where the expression of at least one of these proteins could be maintained or repressed. We started with the *scar*-inducible knockout (SIKO), in which *scar* is expressed from a *tet* promoter in *scar*-null cells (King et al., 2010), giving cells whose SCAR expression is controlled by DOX. Fig. 3 A shows that both SCAR and WASP are located as expected in this strain—SCAR-GFP was found at the tips of pseudopods, whereas GFP-WASP was confined to sites of clathrin-mediated endocytosis when SCAR was present and replaced SCAR at the tips of pseudopods when *scar* was repressed. To create an inducible SCAR/WASP double mutant, *was*A was disrupted in the SIKO background. Clones were maintained in the presence of DOX, so growing cells were *scar*^+^; DOX was removed only shortly before screening. The successful isolation and validation of a double mutant is demonstrated in Fig. 3 B. As in the SIKO parent, SCAR expression was maximally suppressed 48 h after DOX removal, yielding cells deficient in both SCAR and WASP. A low level of SCAR remained after 48 h (Fig. 3 C), but plainly not enough to support growth.

It was immediately apparent that the loss of both SCAR and WASP severely impaired cell growth, explaining why WASP could not be knocked out in a *scar^−^* background (Fig. 3 D). Whereas the loss of SCAR or WASP alone had no effect on growth, the growth of the SIKO/*wasp*^−^ mutant completely arrested 48–60 h after removal of DOX. Because full repression of SCAR took a minimum of 48 h without DOX, this demonstrates the absolute requirement for at least one of SCAR or WASP for *Dictyostelium* growth. Despite this dramatic arrest of growth, cell death was not observed, with cells lacking both SCAR and WASP remaining adherent and capable of readhering if suspended. However, macropinocytosis was completely lost in these cells (Fig. S3 A)—thus one of either SCAR or WASP is needed for macropinocytosis, presumably for forming of functional macropinocytic cups, which are actin- and Arp2/3 complex–based structures (Insall et al., 2001; Veltman et al., 2016).

### WASP family members are required for pseudopod- and bleb-based migration

**Figure 4.**
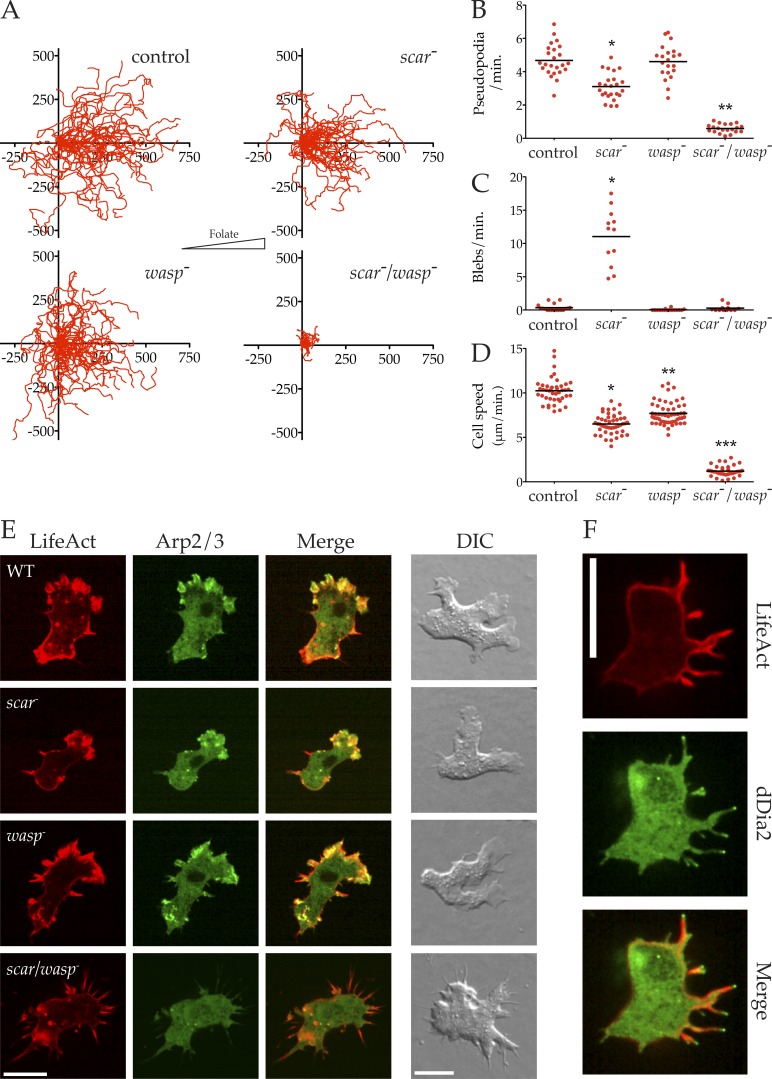
**Cells lacking SCAR and WASP cannot migrate or make pseudopods. (A)** The indicated cells were allowed to chemotax to folate (right) under agarose, followed by phase-contrast microscopy, and tracked as in Fig. 1 B (>20 cells/line from three independent assays; folate gradient oriented toward right; scale is in micrometers). **(B)** Rate of pseudopod formation. Pseudopods were counted from high-magnification DIC videos (60× oil immersion NA = 1.4). Pseudopod production is nearly abolished in double mutant (0.59 ± 0.058 vs. 4.68 ± 0.20 pseudopods/min, mean ± SEM, *n* > 20; P < 0.0001, one-way ANOVA). **(C)** Rate of bleb formation, showing that *scar/wasp* knockout cells lose the blebs that replace pseudopods in *scar* knockouts (0.29 ± 0.14 vs. 11.0 ± 1.2 blebs/min, mean ± SEM, *n* = 12; P < 0.0001, one-way ANOVA; control vs. *scar/wasp* not significant). **(D)** Diminished speed in *scar/wasp* knockout cells compared with control or single mutants. Speeds were derived from tracks in A, showing a decrease in double knockout cells compared with others (7.91 ± 0.22 vs. 6.51 ± 0.16 vs. 7.70 ± 0.17 vs. 1.21 ± 0.096 µm/min, mean± SEM; all values significantly different, one-way ANOVA, Tukey’s multiple comparison; *scar/wasp* vs. *scar* P < 0.0001, unpaired Student’s *t* test; *n* > 40). **(E)** Loss of recruitment of Arp2/3 complex, but not F-actin, to the edges of *scar/wasp* double mutant cells. Cells as indicated expressing LifeAct-mRFP (red) and GFP-ArpC4 (green) were imaged by spinning disc microscopy (Andor Revolution XD) or wide-field DIC. Bar, 5 µm. **(F)** The F-actin spikes in double *scar/wasp* mutant cells are organized by dDia2. Cells expressing LifeAct mRFP (red) and GFP-dDia2 (green) were imaged by spinning disc microscopy (Andor Revolution XD). Bar, 5 µm.

We tested whether cell motility could survive the loss of both SCAR and WASP. By including or excluding DOX from the medium 48 h before any given experiment, we could compare the loss of all combinations of SCAR and WASP—control (SIKO + DOX), *scar*^−^ (SIKO − DOX), *wasp*^−^ (SIKO/*wasp*^−^ + DOX), and *scar^−^*/*wasp*^−^ (SIKO/*wasp*^−^ − DOX). We followed growing cells chemotaxing toward folate (Laevsky and Knecht, 2003), because the developmental defects of mutants without WASP made cAMP chemotaxis unreliable. Tracking cell movement yielded the directional plots shown in Fig. 4 A. Cell migration and chemotaxis are clearly robust in control, *scar*^−^, and *wasp*^−^ cells, but the extremely stunted tracks of the *scar^−^*/*wasp*^−^ cells indicate a severe migration defect (Video 5).

During chemotaxis, *Dictyostelium* cells migrate using pseudopods or blebs or a combination of both, depending on local conditions. Although SCAR knockout cells migrate efficiently, they show a reduced rate of pseudopod formation, which is compensated for by increased blebbing (Veltman et al., 2012). We therefore quantified the number of pseudopods and blebs extended by each genotype during chemotaxis. Control cells primarily migrated using pseudopods, and formed blebs infrequently (Fig. 4, B and C). As expected, *scar*^−^ cells extended significantly fewer pseudopods (Fig. 4 B) and more blebs (Fig. 4 C). Together these were sufficient to maintain cell motility, albeit at a significantly reduced speed (Fig. 4 D). *wasp*^−^ cells were also slightly but significantly slower than control cells (Fig. 4 D). Because *wasp*^−^ cells extended pseudopods at a wild-type rate, it was unsurprising that they formed blebs at normal rates (Fig. 4 C).

However, the *scar^−^*/*wasp*^−^ cells extended virtually no pseudopods (Fig. 4 B). This confirms that the residual pseudopods of *scar^−^* cells depend on WASP (Veltman et al., 2012). Based on the compensatory blebbing observed after the loss of SCAR alone, we expected *scar^−^*/*wasp*^−^ cells to switch entirely to bleb-based motility. However, to our great surprise, *scar^−^*/*wasp*^−^ cells were also unable to form blebs, rendering them essentially immobile (Video 5 and Fig. 4, C and D).

We examined the mechanism that catalyzed actin polymerization in the absence of SCAR and WASP. In contrast to control, *scar^−^*, and *wasp*^−^ cells, the F-actin protrusions in *scar^−^*/*wasp*^−^ cells contained essentially no Arp2/3 complex (Fig. 4 E). Instead, *scar^−^*/*wasp*^−^ cells extended excessive numbers of aberrant F-actin spikes that resembled filopods, with VASP (Fig. S3 B) and the Diaphanous-related formin, dDia2 (Schirenbeck et al., 2005), at their tips (Fig. 4 F).

We conclude that at least one of SCAR and WASP is essential for pseudopods in *Dictyostelium*. Only WASP is capable of substituting for SCAR, and in the absence of both, cells are unable to recruit the Arp2/3 complex to create F-actin protrusions. Remarkably, although loss of Arp2/3 localization made cells much slower, the direction of such migration as remained could be accurately steered by chemoattractants. This conflicts with many theories of chemotaxis, though it agrees with the recent demonstration of chemotaxis in cells that lack the Arp2/3 complex (Wu et al., 2012). In a pseudopod-centered view (Insall, 2010), this result is unsurprising—anything that provides a bias to actin-based motility, whether a local change in growth rate or retraction (Bosgraaf and Van Haastert, 2009), is sufficient to steer cells if protrusions are randomly generated.

However, the unexpected finding that WASP family members are also required for blebbing, which is not thought to involve the Arp2/3 complex, remained unexplained. We therefore investigated the role of SCAR and WASP in bleb formation.

### Normal F-actin levels in *scar*/*wasp*-deficient cells

**Figure 5.**
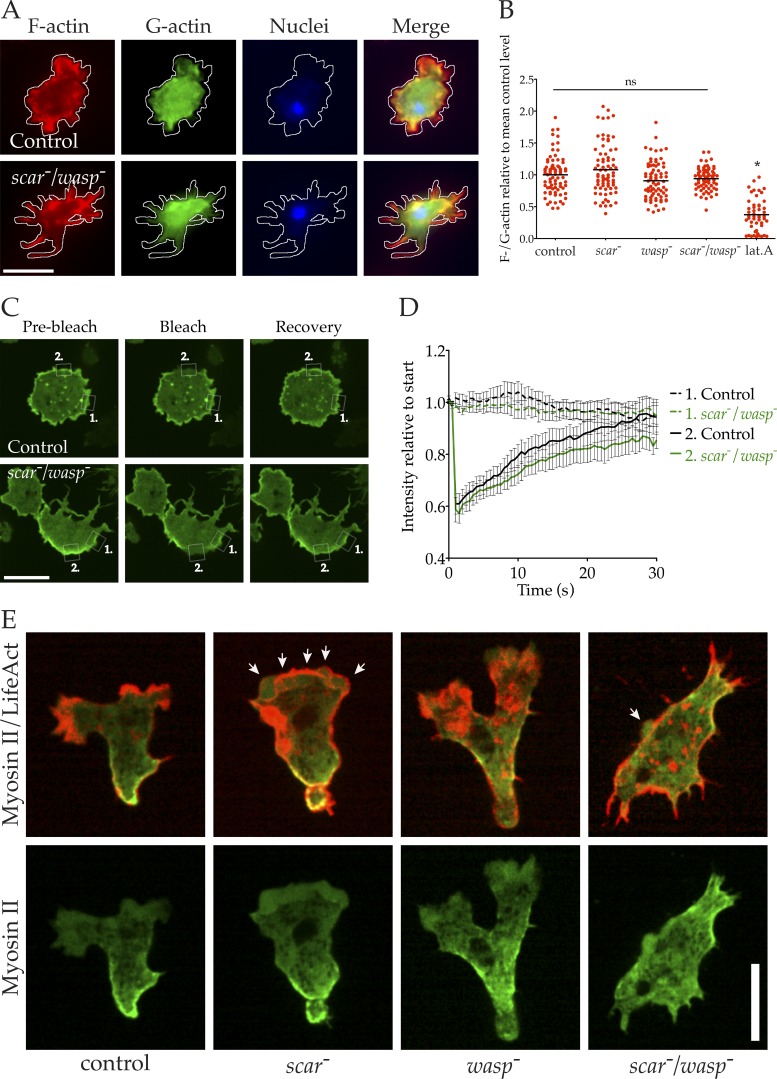
**Dynamic but mislocalized actin in *scar/wasp* mutant cells. (A and B)** F-actin content in *scar/wasp* mutant cells. Cells were fixed and stained with phalloidin (F-actin, red), Dnase I (G-actin, green) and DAPI (DNA, blue) and imaged using wide-field microscopy. **(A)** Control and *scar/wasp* mutant cells fixed and stained with these dyes. Bar, 10 µm. **(B)** F-/G-actin ratios were calculated from many images like those shown in A (*n* = 75) and normalized to the control mean. None of the mutants differed significantly in mean F-actin content compared with the control (one-way ANOVA, Tukey’s multiple comparison). Latrunculin A treatment of control cells significantly reduced mean F-actin content compared with untreated controls (one-way ANOVA, Tukey’s multiple comparison; P < 0.05). C, D. Cortical actin turnover in *scar/wasp* mutants measured by FRAP. **(C)** GFP-actin was expressed in control and *scar/wasp* mutant cells, and FRAP of cortical regions was performed (Andor mosaic FRAPPA unit). White boxes highlight cortical regions measured in **(D)** (region 1 = unbleached control area, region 2 = photo-bleached area). Images represent 1 s before, 0 s, and 30 s after photo-bleach. Bar, 10 µm. **(D)** FRAP curves derived from many cells as shown in B (*n* = 16), with mean fluorescence intensity of regions normalized to initial value; bars = SEM. **(E)** Cortical actomyosin in *scar/wasp* double mutants. Indicated cells expressing LifeAct-mRFP (red) and GFP-MHC (green) were imaged by spinning disc microscopy (Andor Revolution XD). White arrows indicate blebs; lat.A., latrunculin A. Bar, 10 µm.

Because SCAR and WASP are potent promoters of actin polymerization, we tested the possibility that *scar^−^*/*wasp*^−^ cells had a generally compromised cytoskeleton rather than a specific defect in bleb formation. We first investigated whether the inability of *scar*^−^/*wasp*^−^ cells to form blebs was a result of reduced overall F-actin content. Control, *scar^−^*, *wasp*^−^, and *scar^−^*/*wasp*^−^ cells were fixed and costained for F- and G-actin. Again, *scar*^−^/*wasp*^−^ cells formed excessive filopods (Fig. 5 A). Relative F-/G-actin ratios were calculated using phalloidin and DNaseI binding (Bear et al., 1998), and surprisingly, no significant difference was found between control, *scar*^−^, *wasp*^−^, or even *scar*^−^/*wasp*^−^ cells (Fig. 5 B). This contrasts with the reduced F-actin levels seen in cells briefly treated with the actin-depolymerizing drug latrunculin A.

Although *scar*^−^/*wasp*^−^ cells retained normal levels of F-actin, it remained possible that this actin lacked the dynamic behavior of the actin of wild-type cells. To test whether a loss of actin dynamics caused the defect in bleb formation, we expressed GFP-actin and measured the turnover of the actin cortex using fluorescence recovery after photobleaching (FRAP). Fluorescence recovery at the actin cortex (and therefore actin turnover) was evident in both control and *scar*^−^/*wasp*^−^ cells (Fig. 5 C). When the results of many such experiments were plotted, little difference in FRAP was observed between control and *scar*^−^/*wasp*^−^ cells (Fig. 5 D). Also, *scar*^−^/*wasp*^−^ cells were not resistant to latrunculin A, which sequesters G-actin but does not actively depolymerize F-actin (Coué et al., 1987), and were capable of rapidly repolymerizing their cytoskeletons once they were removed from latrunculin A (Video 6). Together, these data demonstrate that *scar*^−^/*wasp*^−^ cells retain a normally dynamic actin cytoskeleton, and actin can be rapidly repolymerized next to the membrane without Arp2/3 complex regulators at the cortex.

We tested other aspects of the cytoskeleton in *scar*^−^/*wasp*^−^ cells including myosin II localization and functionality. Myosin II is found at the rear of migrating cells, where it drives retraction of the trailing tail of the cell. Actomyosin-based contractility is also essential for bleb-based motility (Yoshida and Soldati, 2006; Lämmermann and Sixt, 2009). GFP–myosin II localized to the rear of half of cells during control pseudopod-based motility and *scar*^−^ bleb-based motility (Fig. 5 E). As shown earlier, loss of WASP perturbs tail retraction and myosin II organization. Despite this, *scar*^−^/*wasp*^−^ cells were still capable of recruiting myosin II to the cell cortex, albeit sometimes patchily and without strong front–rear polarity (Fig. 5 E). Furthermore, *scar*^−^/*wasp*^−^ cells were found to retain robust actomyosin-based contractility as provoked by the ATP-depleting poison sodium azide (Fig. S3 C), a well-established test of *Dictyostelium* myosin II function (Patterson and Spudich, 1995).

Together these data demonstrate that *scar*^−^/*wasp*^−^ cells possess a robust and dynamic actin cytoskeleton, so the defect in bleb formation must have another cause.

### *scar*^−^/*wasp*^−^ cells are physically competent to form blebs

**Figure 6.**
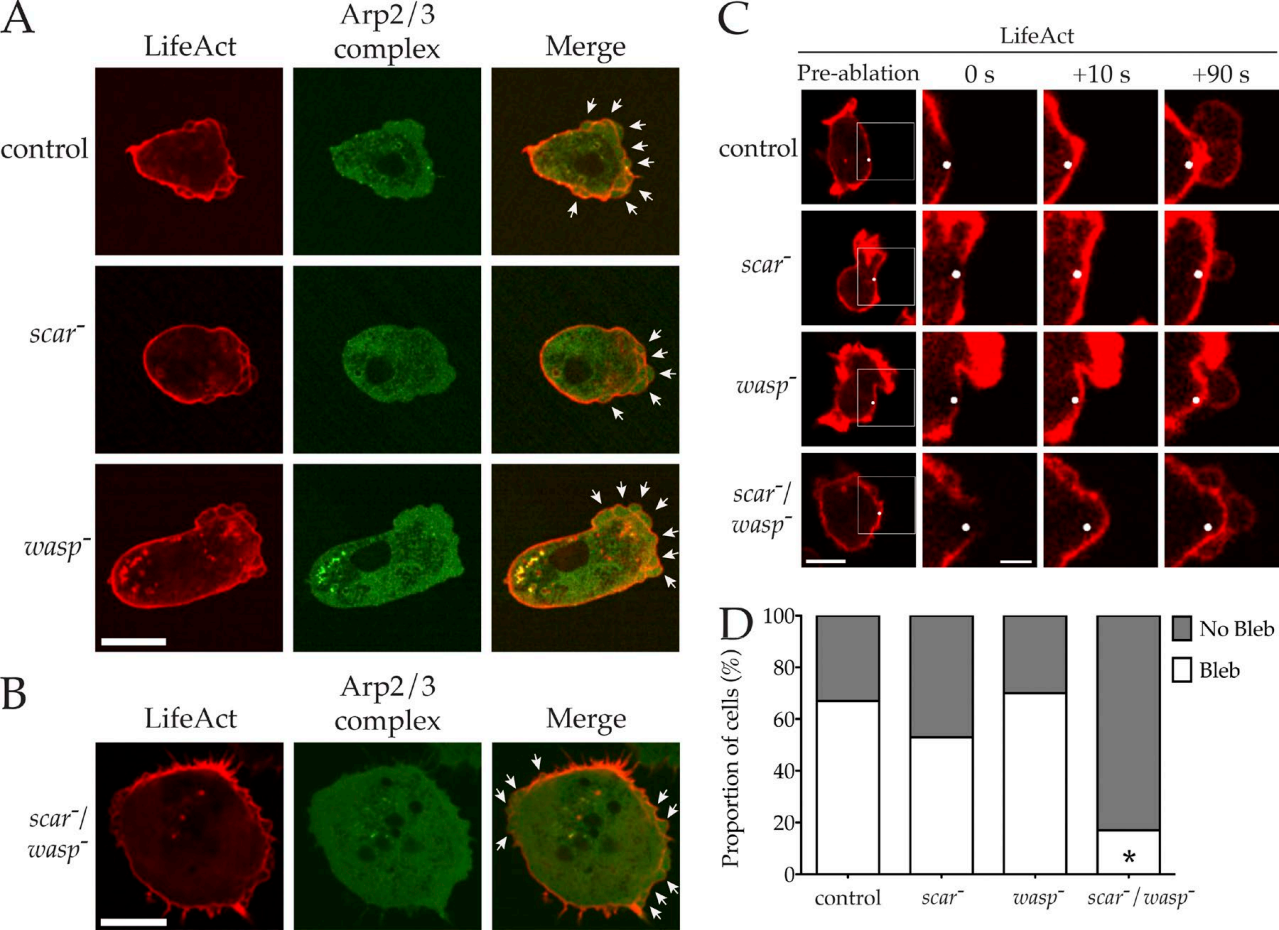
***scar/wasp* mutant cells retain the ability to make blebs. (A)** Cells of indicated genotype expressing LifeAct-mRFP (red) and GFP-ArpC4 (green) were imaged by spinning disc microscopy (Andor Revolution XD) during chemotaxis while highly compressed. White arrows highlight blebs. Bar, 10 µm. **(B)** Highly compressed *scar/wasp* mutant cell expressing LifeAct-mRFP (red) and GFP-ArpC4 (green), imaged using spinning disc microscopy (Andor Revolution XD). White arrows indicate blebs. Bar, 10 µm. **(C and D)** Laser ablation of actin cortex induced blebbing in uncompressed cells. **(C)** Indicated cells expressing LifeAct-mRFP shown before, 0 s, 10 s and 90 s after laser ablation. White dot indicates site of ablation. White box in preablation image enlarged in subsequent post-ablation images. Bars, 10 µm (left showing whole cells); 4 µm (insets). **(D)** Percentage of cells for each genotype that blebbed in response to laser ablation of the cortex (*n* = 30). Only *scar/wasp* double mutants significantly differed from the controls (chi-square test, P < 0.0001) but still induced blebbing ∼20% of the time.

WASP family members are not recruited to the sites of bleb formation, and blebs do not normally appear to require either SCAR or WASP, or to use the Arp2/3 complex (Fig. 3 A). Therefore, we tested whether *scar*^−^/*wasp*^−^ cells were physically capable of forming blebs. Our under-agarose chemotaxis assay strongly favors pseudopod-based migration, with blebbing evident only in *scar*^−^ cells, where pseudopods are reduced (Fig. 4, B and C). We modified conditions to increase compression, which favors bleb-based motility at the expense of pseudopods (Zatulovskiy et al., 2014). Under these conditions, control, *scar*^−^, and *wasp*^−^ cells expressing GFP-labeled Arp2/3 complex and LifeAct-mRFP switched to bleb-based motility (Fig. 6 A). Again, there was no recruitment of the Arp2/3 complex to the site of nascent blebs. Because *scar*^−^/*wasp*^−^ cells were incapable of dragging themselves under a slab of agarose, we compressed stationary cells by placing an agarose sheet directly on them (King et al., 2011). Surprisingly, this induced robust blebbing around the entire periphery of the *scar*^−^/*wasp*^−^ cells (Fig. 6 B).

We also directly induced blebbing in *scar*^−^/*wasp*^−^ cells. Localized laser ablation of the actin cortex has been used to induce the formation of blebs in mouse fibroblasts (Tinevez et al., 2009). We optimized this technique for use with *Dictyostelium* and found we could reproducibly promote bleb formation in uncompressed cells. Laser treatment induced new blebs in all cell types, including *scar*^−^/*wasp*^−^ cells (Fig. 6 C), confirming that the double mutants were still competent to form blebs. Quantification of many such experiments demonstrated that *scar*^−^/*wasp*^−^ cells extend blebs less often than in other cells, after around 20% of laser pulses (Fig. 6 D), perhaps because of a combination of the cells’ thick cortex and lower cortical tension because of slower migration, but those blebs that form are clearly morphologically and behaviorally normal.

Finally, throughout the course of all the experiments outlined in this paper, *scar*^−^/*wasp*^−^ cells were sometimes observed spontaneously blebbing (e.g., in Fig. 5 E). We concluded that WASP family members are not directly required for bleb formation. Instead, *scar*^−^/*wasp*^−^ cells must be indirectly blocked from initiating bleb-based motility.

### Blebs are restored and migration improved in *scar*^−^/*wasp*^−^ cells when dDia2 is also disrupted

**Figure 7.**
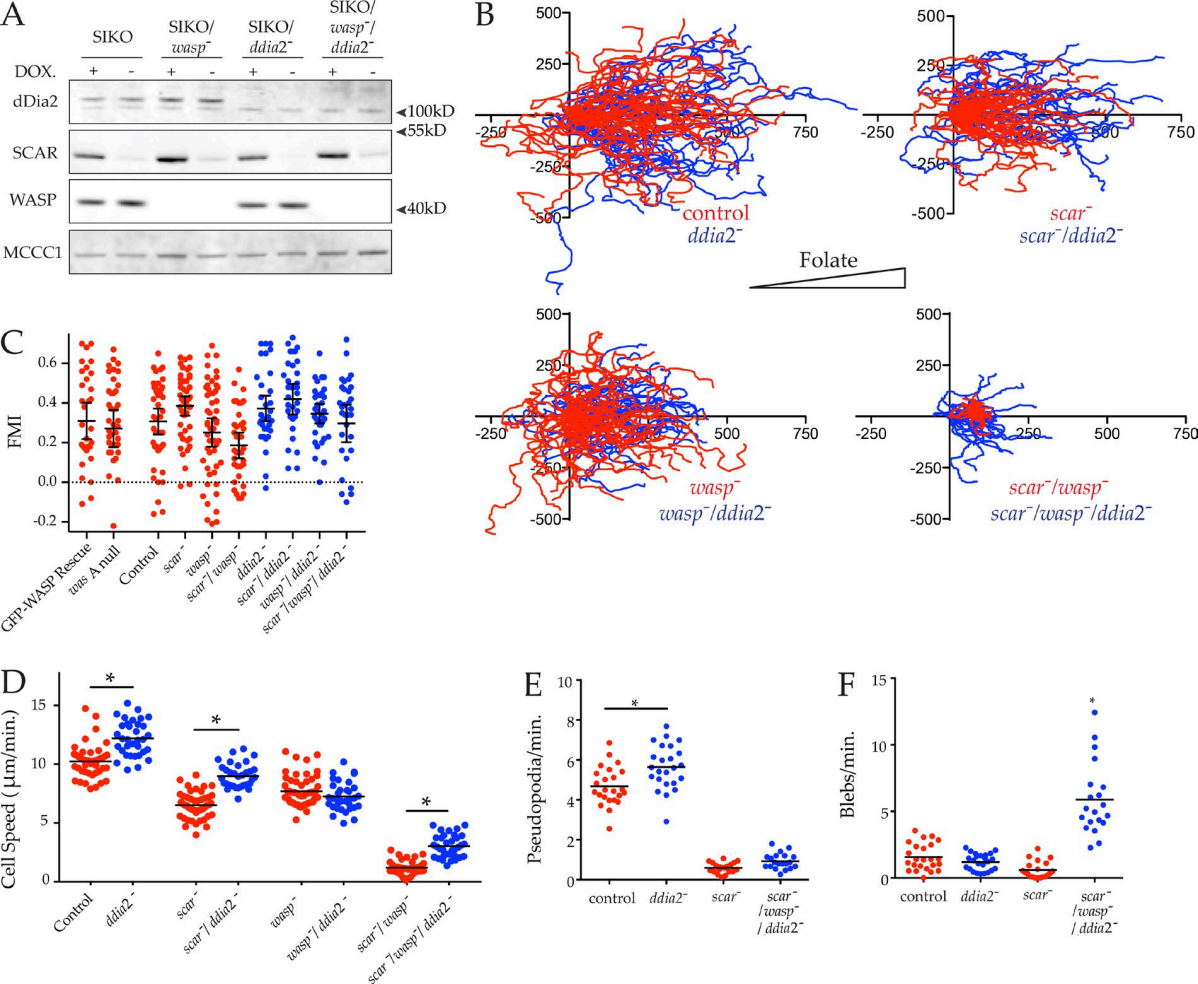
**Loss of a formin, as well as SCAR and WASP, restores movement. (A)** Loss of dDia2, SCAR, and WASP from SIKO/*wasp^−^*/*ddia2^−^* triple mutant cells verified by Western blotting with anti-dDia2, anti-SCAR, and anti-WASP antibodies. Expression of SCAR was suppressed after 48 h in absence of DOX. Biotin conjugates were used to probe for MCCC1 to demonstrate equal loading. (B–E) Motility of *scar/wasp/ddia2* knockouts is improved compared with *scar/wasp* knockouts. The effect of *ddia2* loss in different genetic backgrounds (blue) plotted alongside data from Fig. 4 (A–D) (red) for comparison. **(B)** The indicated cells were allowed to chemotax to folate (right, indicated by triangle) under agarose, imaged by phase-contrast microscopy, and tracked as in Fig. 1 B (*n* > 30 cells/line, three independent experiments). Track length of *scar/wasp/ddia2* knockouts were increased compared with *scar/wasp*; scale is in micrometers. **(C)** Forward migration index (FMI) of tracks from B. All cells were positively chemotactic. **(D)** Speeds were derived from tracks in B. Loss of *ddia2* alone, in a *scar^−^* background or a *scar^−^/wasp^−^* background, significantly increased speed compared with control (12.20 ± 0.25 vs. 10.24 ± 0.22), *scar^−^* (8.98 ± 0.17 vs. 6.51 ± 0.16), or *scar^−^/wasp^−^* cells (3.03 ± 0.16 vs. 1.21 ± 0.10), respectively (all speeds μm/min, mean ± SEM; all values significantly different, one-way ANOVA, Tukey’s multiple comparison, P < 0.05). **(E and F)** Improved motility of *scar/wasp/ddia2* knockouts as a result of increased blebbing as opposed to pseudopod extension. Cells were imaged while chemotaxing under agarose toward folate by DIC. **(E)** Rate of pseudopod formation. DIC videos were analyzed and pseudopods counted. Pseudopod formation was significantly increased in *ddia2^−^* cells compared with controls (5.65 ± 0.23 vs. 4.68 ± 0.20), but no difference was detected between *scar/wasp/ddia2* and *scar/wasp* knockouts (0.93 ± 0.09 vs. 0.59 ± 0.06; all rates pseudopods/min, mean ± SEM, one-way ANOVA, Tukey’s multiple comparison, P < 0.05). **(F)** Rate of bleb formation. DIC videos were analyzed and blebs were counted. The rate of blebbing was significantly increased in *scar/wasp/ddia2* knockouts compared with controls (5.88 ± 0.60 vs. 1.57 ± 0.20), *ddia*2*^−^* cells (1.19 ± 0.13) and *scar^−^/wasp^−^* cells (0.60 ± 0.14; all rates pseudopods/min, mean ± SEM, one-way ANOVA, Tukey’s multiple comparison, P < 0.05).

Because *scar*^−^/*wasp*^−^ cells are capable of forming blebs but seem unable to use them to migrate, we hypothesized that the excessive filopod-like spikes extended by *scar*^−^/*wasp*^−^ cells were obstructing motility. To test this hypothesis, we additionally disrupted *ddia*2 in both the SIKO and the SIKO/*wasp*^−^ background. This yielded mutants where SCAR expression could be suppressed in the absence of dDia2 (*scar*^−^/*ddia*2^−^ cells) or both dDia2 and WASP (*scar*^−^/*wasp*^−^/*ddia*2^−^ cells). These mutants were verified by Western blotting after 48 h in the absence of DOX, which was again able to strongly suppress SCAR expression in all genetic backgrounds (Fig. 7 A).

We then examined how the loss of *ddia*2 affected under-agarose chemotaxis in these cells. Loss of *ddia*2 had little effect on cell migration in the presence of SCAR and WASP, and the additional loss of *ddia*2 did not greatly affect the motility of *scar*^−^ or *wasp*^−^ single mutants. However, strikingly, the loss of *ddia*2 in the *scar*^−^/*wasp*^−^ background partially restored cell motility (Fig. 7 B), yielding substantially longer cell tracks than the *scar*^−^/*wasp*^−^ cells, though chemoattractant-induced actin assembly at the cortex was still completely lost (Fig. S3 D); as with the simple *scar^−^*/*wasp^−^* mutants, the migration was efficiently steered by chemoattractant (Fig. 7 C). Quantitative analysis confirms that *ddia*2^−^/*scar*^−^/*wasp*^−^ cells chemotaxed significantly faster than *scar*^−^/*wasp*^−^ cells (Fig. 7 D). Interestingly, the mean migratory speed of both control and *scar*^−^ cells was also slightly increased after *ddia*2 loss. However, this was not true for *ddia2*^−^/*wasp*^−^ cells, which migrated at the same speed as *wasp*^−^ cells.

We next examined the restored motility of *ddia*2^−^/*scar*^−^/*wasp*^−^ cells in detail using high-magnification differnetial interference contrast (DIC) microscopy, allowing the pseudopods and blebs to be quantified. The *ddia*2*^−^*/*scar^−^*/*wasp^−^* cells tolerated LifeAct expression poorly, with few expressing cells. Rates of bleb formation were therefore compared using DIC videos. Compared with the controls, *ddia*2^−^ cells extended pseudopods slightly more rapidly, which likely accounts for their increased speed (Fig. 7 E). However, pseudopods were no more evident in *ddia*2^−^/*scar*^−^/*wasp*^−^ cells than *scar*^−^/*wasp*^−^ cells, consistent with WASP family members having an essential role in pseudopod formation. Because loss of *ddia*2 alone stimulated pseudopod formation, it was not surprising to find that *ddia*2^−^ cells blebbed infrequently (Fig. 7 F). However, in contrast to *scar*^−^/*wasp*^−^ cells, *ddia*2^−^/*scar*^−^/*wasp*^−^ cells were able to generate new blebs, and thus regained significant cell migration (Fig. 7 F).

**Figure 8.**
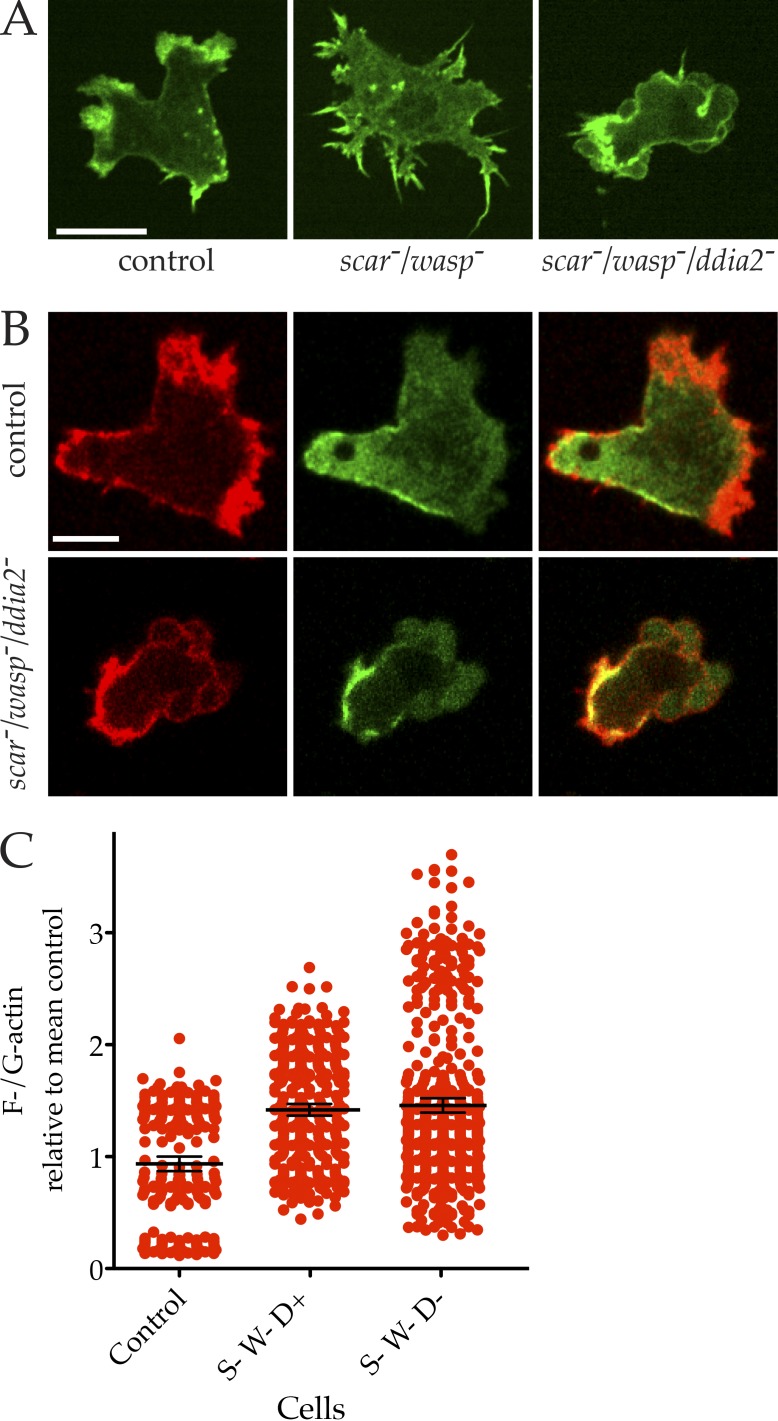
**Actin and myosin II in *scar^−^/wasp^−^* cells with and without *ddia2*. (A)** *scar/wasp/ddia2* knockouts chemotax through bona fide blebs. Cells expressing LifeAct-GFP were imaged chemotaxing under agarose by spinning disc microscopy (Andor Revolution XD). Compared with the pseudopods of chemotaxing controls and the filopods extended by immobile *scar/wasp* knockouts, the motility of *scar/wasp/ddia2* is supported by blebbing. Bar, 10 µm. **(B)** Myosin II in *scar/wasp* and *scar/wasp/ddia2* mutants. Cells were transfected with GFP–myosin II heavy chain and RFP-LifeAct, and imaged by confocal. Myosin II is clearly localized at the rear, with blebs at the front of *scar/wasp/ddia2* mutant cells. Bar, 5 µm. **(C)** F-actin content in *scar/wasp/ddia2* mutant cells (labeled S, W, and D). Cells were fixed and stained with Texas red phalloidin (F-actin) and Alexa Fluor 488 DNase I (G-actin) and imaged using confocal microscopy. Summed projections were made and the ratios of F-/G-actin calculated from many images (*n* = 75) and normalized to the control mean. None of the mutants differed significantly in mean F-actin content compared with the control (one-way ANOVA, Tukey’s multiple comparison).

The improvement in migration in *scar*^−^/*wasp*^−^ cells after *ddia*2 was also knocked out corresponded with a clear return in cell polarity (Fig. 8 A) and myosin II localization (Fig. 8 B and Video 7); although the *ddia*2^−^/*scar*^−^/*wasp*^−^ cells are by no means normal, they lack the frozen appearance of the double *scar*^−^/*wasp*^−^ cells. The myosin II localization appears to follow the position of the blebs, rather than anticipating it (Video 7), implying that myosin does not determine the blebs’ position.

Most surprisingly, the relative amount of F-actin was not substantially diminished (Fig. 8 C)—although the cells grew unevenly and variation between experiments became substantial (and poor LifeAct expression suggests their cytoskeletons were too damaged to tolerate another actin-binding protein), the trend was for the F-actin level to remain constant, or even grow slightly. Thus, remarkably, cells can maintain their G-actin/F-actin ratio even when three main nucleation regulators are lost.

We conclude that WASP family members and the Arp2/3 complex normally compete with dDia2 and other formins to induce new actin polymerization at the cortex. This dynamic competition acts to maintain a plastic actin cytoskeleton. However, unconstrained activity of dDia2 in the absence of competition from SCAR and WASP results in excessive actin spike formation, which is incompatible with blebbing in particular and cell motility in general.

## Discussion

We currently have a growing appreciation of how subsets of actin regulators come together to form individual actin-based structures. However, complex cell behavior such as cell motility and chemotaxis requires cells to deploy highly choreographed combinations of different actin-based structures. Exactly how cells spatially and temporally coordinate the activities of these different actin-assembly factors remains poorly understood. Recently, competition for actin monomers has emerged as a means by which different actin nucleators can influence each other’s activities and therefore the type of structures generated (Rotty and Bear, 2014; Lomakin et al., 2015; Davidson and Wood, 2016; Suarez and Kovar, 2016). Here we have built upon these findings and have demonstrated an important role for cytoskeletal competition in cell motility.

### WASP family proteins are essential for pseudopod formation

We generated mutants devoid of WASP. We clearly show that WASP is not required for pseudopod formation, or for macropinocytic growth in liquid medium. On bacteria, the cells lacking WASP grew very poorly. We have not found whether this reflects a defect in phagocytic engulfment of particles, or a failure to sort the enzymes needed to digest the more complex food source as seen with mutants in WASH complex genes (Buckley et al., 2016), but because WASP localizes to phagocytic cups, it seems more likely the issue is with engulfment.

We also generated cells lacking both SCAR and WASP, confirming that WASP is the only nucleation promoting factor making the residual pseudopods extended by the *Dictyostelium scar* null. Importantly, no other proteins are capable of recruiting the Arp2/3 complex to the cortex of cells to promote pseudopod formation. Replacement of SCAR/WAVEs by WASPs is seen to different degrees in other organisms. In nematodes it is clear (Zhu et al., 2016); in mammalian cells, N-WASP is required for the invasion of mammalian cells through a 3D matrix (Tang et al., 2013), but it is difficult to disentangle whether this is because it has a role in normal 3D pseudopods, or because it is required for clathrin-mediated endocytosis and normal secretion of matrix metalloproteinase, which are required for migration through complex environments. Importantly, the excessive filopods extended by the *Dictyostelium scar*/*wasp* mutant resemble the phenotype observed when SCAR or the Arp2/3 complex is disrupted in other organisms (Kunda et al., 2003; Zhu et al., 2016), which supports the hypothesis that competition between Arp2/3 regulators and formins is conserved.

Unlike in previous studies (Suraneni et al., 2012; Wu et al., 2012; Rotty and Bear, 2014), here the Arp2/3 complex was not globally inhibited or lost, but instead could not be recruited to the cortex in the absence of SCAR or WASP. The actin-assembly factors underlying the increase in actin bundles observed after Arp2/3 complex disruption vary depending on the organism studied. Here, we have found that they are the product of the Diaphanous-related formin, dDia2, consistent with its established role in normal *Dictyostelium* filopod formation (Schirenbeck et al., 2005).

Although cell growth was very quickly arrested in the absence of both SCAR and WASP, these mutants retained a surprisingly robust cytoskeleton. This was most evident in their ability to completely reform their actin cytoskeleton when released from latrunculin inhibition. *scar*/*wasp* mutant cells were fully capable of reconstituting their cytoskeleton despite their inability to use the Arp2/3 complex. They are also able to orient themselves, despite their serious defects in actin, in agreement with Wu et al. (2012), who found that the Arp2/3 complex was not essential for chemotaxis. We imagine that multiple pathways robustly use different mechanisms converging on chemotactic steering (Insall, 2013). Thus loss of any one player, even one that is centrally important for normal movement like Arp2/3 complex–driven pseudopod formation, is unable to block all steering.

Surprisingly, but consistently with competition between actin-assembly factors for a finite pool of G-actin, F-actin levels remained constant between the different mutants. In the *scar*/*wasp* mutant, G-actin normally used by the Arp2/3 complex to form dendritic networks is now available to dDia2. This would fuel increased dDia2 activity, resulting in excessive filopod formation and ultimately no net change in F-actin levels. Although *scar*/*wasp* mutants’ mean F-actin content was not greatly changed, the range of values between cells was relatively small. This was consistent with the static morphology seen during live cell imaging of the *scar*/*wasp* mutants. However, our FRAP data imply that the actin of *scar*/*wasp* mutants turns over normally; the low variance in F-actin levels does not reflect nondynamic actin, but the loss of cycles of extension and retraction of pseudopods. The morphology of *scar*/*wasp* mutants appeared trapped in a paralyzed configuration, caused by sustained and unchanging dDia2 activity.

### A balance of Arp2/3 complex and dDia2 activity is required for bleb-based motility

WASP can only partially compensate for loss of SCAR in *Dictyostelium*, and the residual pseudopods of *scar*-deficient cells are supplemented with blebs. SCAR, WASP, and the Arp2/3 complex do not localize to the sites of bleb formation, and we had expected that *scar*/*wasp* mutants would chemotax through the use of blebs alone. However, *scar*/*wasp* mutants failed to make blebs to maintain motility, making them essentially immobile. Crucially, *scar*/*wasp* mutants were physically capable of blebbing either when compressed or when the cellular cortex was laser-ablated. Only after dDia2 was additionally disrupted in the *scar*/*wasp* mutant was bleb- (but not pseudopod-) based migration restored. This confirms that neither the Arp2/3 complex nor dDia2 is required for the reformation of the actin cortex that occurs during blebbing.

The partial suppression of Arp2/3 complex activity found in the *scar* mutant or via specific Arp2/3 complex mutants increases blebbing. However, the total loss of the Arp2/3 complex from the actin cortex also results in excessive dDia2 activity, which overwhelms the actin cytoskeleton and inhibits blebbing. Consistent with previous studies, we also found that disruption of *ddia*2 alone significantly increases speed, suggesting that Diaphanous-related formins also constrain the activity of the Arp2/3 complex. These data imply that the cytoskeletal plasticity evident in *Dictyostelium* and all cells exists when the competing activities of actin-assembly factors are balanced. The mutants studied here represent extreme, global skewing of this competition. However, if cells were capable of influencing this cytoskeletal competition on a subcellular scale, it would allow them to coordinate their actin regulators as a collective, rapidly induce dynamic rearrangements in their actin cytoskeletons, and ultimately promote seamless changes in cell behavior.

## Materials and methods

### Cell culture

*Dictyostelium* Ax3 cells were cultured on petri dishes in HL5 (Formedium^T^). For transfection, cells were washed of HL5 and suspended in E-buffer (10 mM KNaPO_4_, pH 6.1, and 50 mM sucrose), incubated with DNA, and electroporated at 500 V using an ECM 399 Electroporation System (BTX Harvard Apparatus). Cells were then immediately returned to petri dishes containing HL5. Selection and maintenance of extrachromosomal plasmids was achieved through addition of 50 µg/ml hygromycin to the media. Cell growth was measured in the presence of a substratum on petri dishes or in suspension in shaking flasks. Cell counts were performed every 12 h with a CASY Cell Counter + Analyser system Model TT (Innovates AG). For development, cells were washed in development buffer (10 mM KNaPO_4_, 2 mM MgCl_2_, and 1 mM CaCl_2_, pH 6.5) and left to starve on 1% agar at high confluency. Lysates were prepared or images captured every 2 h over the course of development.

### Restriction enzyme–mediated integration (REMI) and knockout generation

To generate the WIKO, *gfp-was*A was cloned into a DOX-inducible expression vector (Veltman et al., 2009). This construct was stably introduced into the *Dictyostelium* genome by REMI (Kuspa and Loomis, 1992; Insall et al., 1994). Selection was performed using 50 µg/ml hygromycin. The inducibility of the isolated clones was verified by treating the cells with or without 10 µg/ml DOX, and GFP-WASP expression was detected by Western blot (antibody was a gift from T. Soldati, University of Geneva, Geneva, Switzerland). The *was*A and *ddia*2 loci were disrupted by homologous recombination using knockout vectors generated by PCR. Clones were screened based on resistance to 20 µg/ml blasticidin (*was*A knockout) or 30 µg/ml nourseothricin (*ddia*2 knockout) and verified by Western blot (anti-dDia2 antibody was a gift from J. Faix, Medizinische Hochschule Hannover, Hannover, Germany). The inducible expression of WASP or SCAR was maintained with 10 µg/ml DOX where appropriate.

### Immunoblotting

Western blotting was performed as previously described (Davidson et al., 2013b). In brief, equal numbers of cells were pelleted and immediately lysed with 70°C 1× NuPAGE LDS sample buffer (Life Technologies) containing 50 mM DTT reducing agent. The lysates were then separated using 10% Bis/Tris NuPAGE polyacrylamide gels (Life Technologies). After SDS-PAGE, proteins were transferred onto nitrocellulose membranes (Hybond-C-extra; Amersham Biosciences) in a BioRad transfer tank filled with 1× SDS-transfer buffer at 100 V for 1 h. The membranes were then blocked with a solution containing 5% nonfat dried skimmed milk dissolved in TBS, after which they were probed with primary antibodies (1:1,000 dilution) overnight at 4°C. Detection was achieved through the use of HRP or fluorescently conjugated secondary antibodies combined with chemiluminescence (Immobilon Western chemiluminescent HRP substrate; Millipore) or an Odyssey IR imaging system (LICOR Biosciences). Coomassie staining or probing for MCCC1 with Alexa Fluor 680–conjugated streptavidin (Davidson et al., 2013a) was used to confirm equal loading.

### Chemotaxis assays

Cell motility was studied through the use of an under-agarose folate chemotaxis assay (Laevsky and Knecht, 2003). Agarose (SeaKem GTG) was melted in SIH medium (Lu et al., 2004) to yield a 0.4% gel, 5 mL of which was cast in a 50-mm glass-bottomed dish (MatTek) that had been pretreated with 10 mg/ml BSA. Once the agarose had set, two 5 × 20-mm wells were cut 5 mm apart in the center of the gel using a scalpel. This left a central “bridge,” which was gently wriggled loose. Vegetative cells were harvested, pelleted by centrifugation (3 min at 380 *g*), and resuspended in SIH medium at 0.5–1 × 10^6^ cells/ml. One well of the assay was filled with cell suspension, whereas the other was filled with 0.01 mM folic acid diluted in SIH medium. A coverslip was then carefully lowered on top of the agarose to cover the majority of the wells and prevent evaporation. The chemotaxis of the vegetative cells under the agarose bridge was monitored, and wild-type cells were found to reach migratory speeds of >10 µm/min. To promote migration through blebs rather than through pseudopods, the concentration of agarose was increased to 1.5% and was cast in untreated glass-bottomed dishes. The severe compression of immobile cells was performed as described (King et al., 2010). In brief, cells were washed in SIH medium and plated in 30-mm glass-bottomed dishes (MatTek). The cells were then compressed under a slab of 1% agarose gel (SeaKem GTG) using an array of metal discs.

For global folate stimulation and assessment of cortical F-actin levels, cells expressing LifeAct-GFP were placed into 8-well chamber slides (thickness 1.5; Lab-Tek) in LoFlo buffer for 90 min, at 10^5^ cells/well. Airyscan imaging was performed, focusing on the actin cortex midway through the cell. Images were acquired at 1-s intervals. After a few frames, folate was added to a final concentration of 1 mM. Images were captured for another 20–30 s. For analysis, an ImageJ (National Institutes of Health) macro was written to segment the cell into plasma membrane and cytosol regions: a mask was made to outline the cell and another to encompass the cytosol. Intensities of each region were assessed before and after folate stimulation. Manual analysis showed that the automated macro was accurate.

### Cell fixation and staining

Cells were seeded at low density on glass coverslips and then fixed for 5 min (6% formaldehyde [wt/vol], 15% picric acid [vol/vol], and 10 mM Pipes, pH 6.5). The cells were washed with PBS before permeabilization with 70% ethanol for 2 min. The fixed cells were washed repeatedly with PBS and then stained for 30 min with 33 nM Texas red phalloidin (Life Technologies) for F-actin. For ratiometric staining, this was combined with 161 nM Alexa Fluor 488 DNaseI (Life Technologies). The coverslips were washed with PBS and dH_2_O before being mounted on glass slides with antifade reagent containing DAPI (Prolong Gold; Life Technologies). For the quantification of multinuclearity, the number of nuclei per cell was counted for 100 s of cells/cell line over multiple experiments.

For ratiometric quantification of F-actin levels, 75 cells/cell line over three experiments were outlined using ImageJ software, and the mean intensity of both the F-actin and G-actin staining within the area of the cell was determined. A F-/G-actin ratio was calculated for each cell, and all the values were the normalized to the mean F-/G-actin ratio of the wild-type control. As a control, wild-type cells were also submaximally (5 min) treated with 5 µM latrunculin A.

### Actomyosin contractility test

Myosin II function was tested by treating cells with sodium azide (Patterson and Spudich, 1995; Xu et al., 2001). Cells were treated with 5 mM sodium azide for 15 min, after which a cell count was performed using a CASY Cell Counter + Analyser system Model TT. The mean proportion of detached cells was calculated for each cell line over four independent experiments.

### Microscopy

Phase contrast and DIC images were acquired through the use of an Eclipse TE2000-E inverted microscope (Nikon) equipped with a monochrome Retiga Exi cooled CCD camera. Phase-contrast microscopy was performed using either a 10×/0.3 NA or a 20×/0.45 NA Nikon Plan Fluo objective. DIC microscopy was performed using either a 60×/1.4 NA or a 100× 1.4 NA Nikon Plan Apo objective and oil immersion. *Dictyostelium* was imaged at 22°C.

Images of fixed and stained cells were acquired through the use of an inverted wide-field microscope (IX81; Olympus) with a 60× 1.42 NA Plan Apochromat objective. This microscope was equipped with a Photometrics Coolsnap HQ camera.

TIRF microscopy was adopted for the imaging of the basal surface of cells and was advantageous for visualizing clathrin-mediated endocytosis in live cells. Dual-color TIRF microscopy was performed simultaneously on a modified Eclipse TE 2000-U microscope in conjunction with a 100×/1.45 NA TIRF objective (Nikon), a photometrics Evolve 512 camera, and a DualView DV2 emission splitter. Images were recorded every second.

Spinning disc confocal microscopy was performed with an Andor Revolution XD spinning disc system (Ti-E inverted microscope [Nikon] with a CSU-X spinning disc confocal unit [Yokogawa] and a high-resolution Neo sCMOS camera [Andor]). This system was used in combination with a 60×/1.4 NA or a 100×/1.4 NA objective. Images were acquired every 2 s. Photobleaching was performed using an Andor mosaic FRAPPA unit. Macropinocytosis was measured exactly as in Thomason et al. (2017).

Cortical ablation was performed using the FRAP unit of a Fluoview FV1000 (Olympus) with a planApo N60×/1.4 oil objective. A single point on the cell cortex was ablated for 8 s with a 405-nm laser, after which images were acquired every 2 s.

Airyscan imaging was performed on a Zeiss 880 inverted confocal microscope using the Super Resolution setting, with either a 40×/1.3 NA or a 63×/1.4 NA objective. Airyscan processing used default settings of deconvolution at strength 6.

### Image processing and statistical analysis

All images acquired by microscopy were exported as TIFFs and imported into ImageJ. Noise was reduced in these images through the use of the despeckle tool in ImageJ. Otherwise the images were cropped and resized and their contrast/brightness was altered. Image analysis was always performed on the raw, unprocessed TIFFs. Often, cells or regions of interest were outlined, and the mean fluorescence intensity was measured within such defined areas. Kymographs were constructed through the reslice tool in ImageJ. The majority of data were generated by analyzing image stacks frame by frame for the appearance of different cellular structures, e.g., pseudopods were identified in DIC images as translucent cellular protrusions extended over multiple frames. The colocalization of the Arp2/3 complex and F-actin was additionally used with confocal images. Blebs were identified in DIC time lapses as near instantaneous bulges of the cell membrane and as lacking Arp2/3 complex localization in confocal images. Unpaired Student’s *t* tests or one-way ANOVA with a Tukey’s multiple comparisons test were used to test statistical significance and generate P values.

### Online supplemental material

Fig. S1 shows generation, growth, and development of WASP knockout cell lines. Fig. S2 shows that the WASP knockout has a cytokinesis defect. Fig. S3 shows cytoskeletal function in *scar^−^/wasp^−^* cells. Video 1 shows pseudopod formation in chemotaxing GFP-WASP/*was*A and *was*A^−^ cells. Video 2 shows actomyosin dynamics in chemotaxing wild-type and wasA^−^ cells. Video 3 shows WASP recruitment at the sites of clathrin pit internalization. Video 4 shows exaggerated tails at the rears of wasA^−^ cells. Video 5 shows lack of movement in *scar/wasp* double mutants. Video 6 shows actin dynamics in *scar/wasp* double mutants. Video 7 shows myosin II localization in triple mutants.

## Supplementary Material

Supplemental Materials

Video 1

Video 2

Video 3

Video 4

Video 5

Video 6

Video 7
