# Supplementary material for: WASP family proteins and formins compete in pseudopod- and bleb-based migration
Source: J Cell Biol. 2018 Feb 5;217(2):701–14. doi: 10.1083/jcb.201705160 (PMC5800805; doi:10.1083/jcb.201705160)
Supplement: Supplemental Materials [file JCB_201705160_sm.pdf]

Davidson et al., https://doi.org/10.1083/jcb.201705160

THE JOURNAL OF CELL BIOLOGY

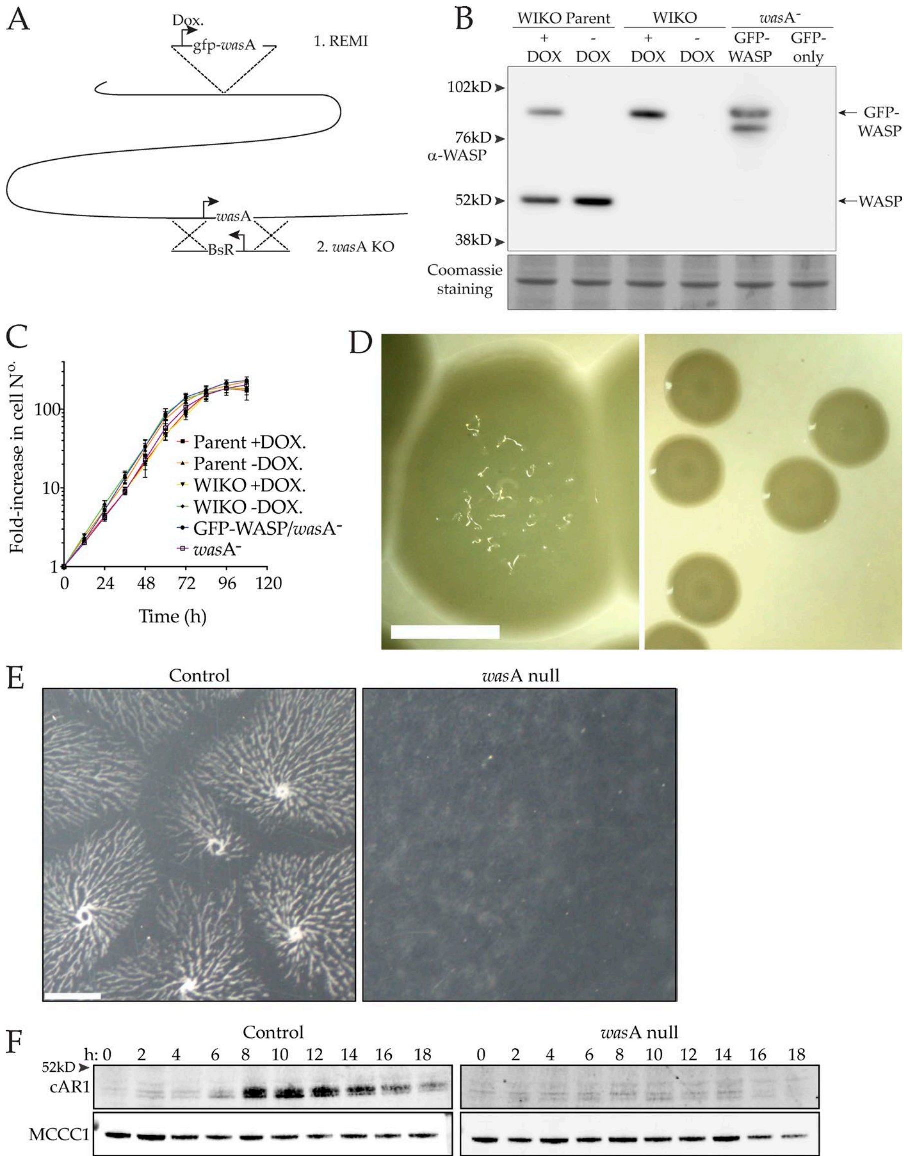

Figure S1. **Generation, growth and development of WASP knockout cell lines. (A)** Diagrammatic representation of WASP knockout strategies. GFP-WASP under the control of a DOX-inducible promoter was introduced into the genome of Ax3 cells by REMI to create the WIKO parent (1). The endogenous *was*A locus was then targeted for deletion by homologous recombinationunder blasticidin selection using the BsR marker to create the WIKO (2). Alternatively, *was*A was targeted for deletion in the Ax3 cells to create the *was*A⁻ mutant. **(B)** Validation of the WASP knockout cell lines by Western blotting. Top: Lysates prepared from the WIKO parent/WIKO ± DOX, and the *was*A⁻ cells ± GFP-WASP were probed with an anti-WASP antibody. The lower band corresponding to endogenous WASP has clearly been lost in the WIKO and the *was*A⁻ cell lines. A double band was always present when GFP-WASP was expressed extrachromosomally and was presumed to be a degradation product resulting from excessive expression. Bottom: Subsequent Coomassie staining of the membrane was used to demonstrate equal loading. **(C)** WASP is not required for *Dictyostelium* cell viability. Growth curves demonstrating that *was*A-deficient cell lines have no defect in growth when cultured on petri dishes. The WIKO parent/WIKO ± DOX and the *was*A⁻ mutant ± GFP-WASP were plated on petri dishes, and a cell count was performed every 12 h over a period of 5 d. The mean fold-increase in cell number was calculated from several independent experiments (WIKO parent/WIKO ± DOX, *n* = 2; *was*A⁻ mutant ± GFP-WASP, *n* = 3) and plotted in the graph. The error bars indicate the SEM. **(D)** Poor bacterial growth of WASP mutants. Individual Ax3 and *was*A⁻ mutant cells were plated on a lawn of *Klebsiella aerogenes* bacteria on SM agar and allowed to grow and phagocytose a plaque for 6 d. The mutant colonies are very much smaller. Bar, 1 cm. **(E)** WASP is required for normal development. Parental Ax3 and *was*A⁻ mutant cells were washed and allowed to adhere to nonnutrient agar, and their development was observed with a dissecting microscope. The mutants failed to initiate development. **(F)** cAMP receptor expression in WASP mutants. Ax3 and *was*A⁻ mutant cells were washed, allowed to adhere to nonnutrient agar, and developed for the indicated number of hours before being harvested, separated, and analyzed by Western blotting. cAR1 is clearly not expressed in mutant cells, rendering them insensitive to extracellular cAMP. Bottom: MCCC1 loading control.

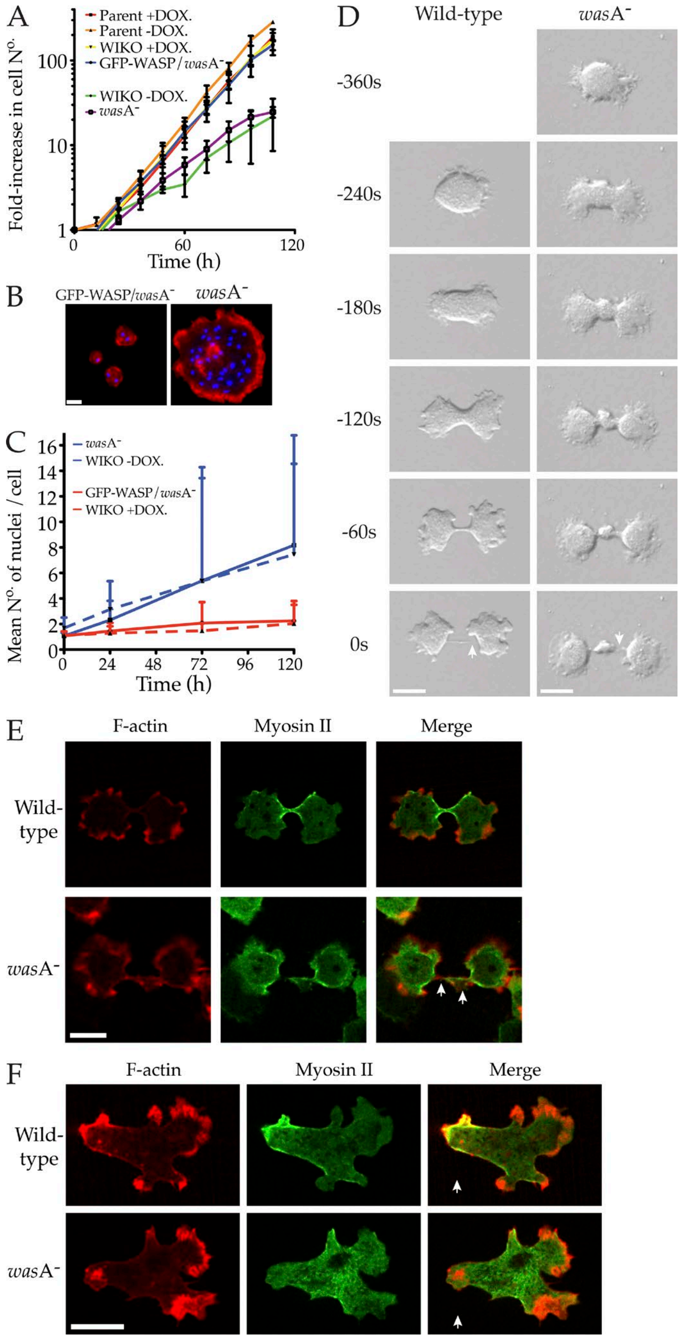

Figure S2. **The WASP knockout has a cytokinesis defect. (A)** WASP-deficient cells grow extremely slowly in suspension. Growth curves demonstrating that *was*A-deficient cells grow very poorly when cultured in suspension. The WIKO parent/WIKO ± DOX and the *was*A⁻ mutant ± GFP-WASP were introduced to shaking culture, and a cell count was performed every 12 h over 5 d. The mean fold-increase in cell number was calculated for each cell line over several independent experiments (WIKO parent/WIKO ± DOX, *n* = 2; *was*A⁻ mutant ± GFP-WASP, *n* = 4) and plotted in the graph. The error bars indicate the SEM. **(B)** The WASP knockout becomes severely multinucleate in shaking culture. An extreme example of a multinucleate *was*A⁻ cell after 120 h shaking culture compared with a GFP-WASP/*was*A⁻ control. Cells were fixed and stained with fluorescently labeled phalloidin (F-actin) and DAPI (Nuclei). Bar, 10 µm. **(C)** WASP deficient cells become progressively more multinucleate with increasing time in suspension. WIKO ± DOX and the *was*A⁻ mutant ± GFP-WASP were introduced to shaking culture. Sample of cells for each cell line were taken at selected time points and were fixed and stained with fluorescently labeled phalloidin (to stain for F-actin) and DAPI (to stain for the nuclei). The number of nuclei per cell was counted at the indicated time points, and the mean was plotted to demonstrate that WASP-deficient cells rapidly become multinucleate when cultured in suspension (*was*A⁻ mutant ± GFP-WASP, $n \approx 200$ cells/cell line over two independent experiments; WIKO ± DOX, $n \approx 100$ cells/cell line for one experiment). The error bars represent SD (only top bars shown for clarity). **(D)** WASP knockout cells dividing on a glass surface exhibit abnormal bulging in the cleavage furrow during ingression. Individual DIC images of representative dividing wild-type and *was*A⁻ cells. Images separated by indicated time (in seconds) before abscission (0 s). The *was*A⁻ mutant takes longer to complete cytokinesis and frequently exhibits a substantial midbody in its cleavage furrow. The white arrows indicate point of abscission. Bars, 10 µm. **(E and F)** Perturbed recruitment of myosin II. Cells were transfected with LifeAct-mRFP and GFP-mhcA and visualized using spinning disc microscopy. **(E)** Cleavage furrow. The localization of myosin II was found to be perturbed in the furrow of dividing *was*A⁻ cells, particularly in the vicinity of the characteristic midbody often formed between the two daughter cells (white arrow). Bar, 10 µm. **(F)** Trailing tail. Myosin II is enriched in the tails (white arrows) of wild-type but not *was*A⁻ cells. Representative cells. Bar, 10 µm.

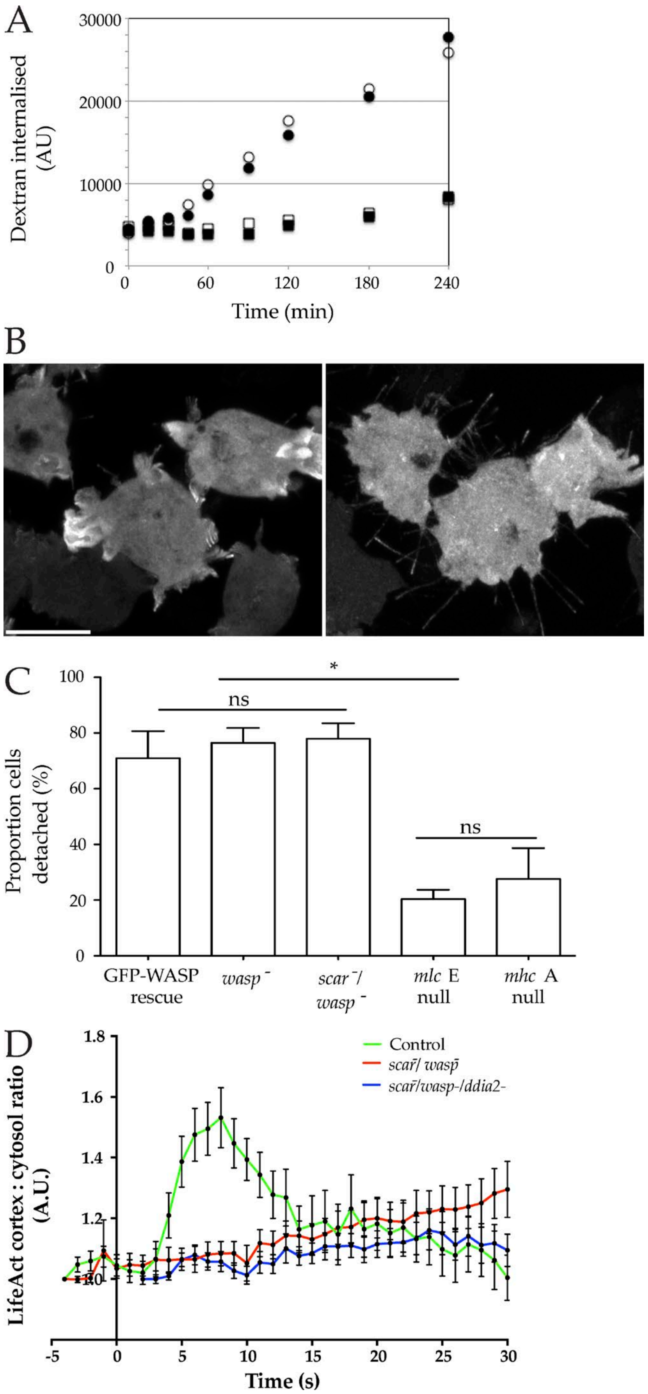

Figure S3. **Cytoskeletal function in *scar*⁻/*wasp*⁻ cells. (A)** Internalization of fluorescent dextran by SIKO/*wasp*⁻ cells grown with (circles) and without (squares) DOX. The dox⁺ cells are phenotypically *scar*⁺/*wasp*⁻, and dox⁻ are *scar*⁻/*wasp*⁻. Simultaneous loss of both SCAR and WASP causes macropinocytosis to drop to negligible levels (representative of three experiments). **(B)** VASP localization in the filopod-like F-actin spikes of *SIKO/wasp*⁻ cells grown with (left) and without (right) DOX. Cells were transfected with GFP-VASP and viewed in a Zeiss Airyscan confocal. Z-projections are shown to accommodate filopods and phagocytic cups. Essentially all spikes terminate with a pronounced GFP-VASP punctum. Bar, 10 μm. **(C)** Robust actomyosin contractility. 5 mM sodium azide induced robust actomyosin-based contraction and detachment in GFP-WASP rescue, *wasp*⁻, and *scar*⁻/*wasp*⁻ cells. In contrast, the proportion of myosin II mutants (*mlc*A and *mhc*A nulls) that failed to detach in response to azide was strongly reduced. *Significant difference between GFP-WASP rescue, *wasp*⁻ and *scar*⁻/*wasp*⁻ cells, and myosin mutant controls. ns, nonsignificance (1-way ANOVA, Tukey's multiple comparison, $P < 0.001$); error bars = SEM. **(D)** Complete loss of attractant-induced actin assembly. Cells were transfected with GFP-LifeAct and imaged every 5 s before, during, and after treatment with homogeneous 10 μM folate. The relative densities of GFP marker present at the periphery and center of the cell were expressed as a ratio, then normalized to 100% at the pretreatment resting state of the cell. The parent shows a pronounced actin polymerization response to folate, whereas no change is perceptible in the mutant whether ddia2 is present or absent (n = 3 experiments for each strain).

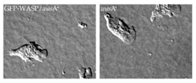

Video 1. **Pseudopod formation in chemotaxing GFP-WASP/*was*A and *was*A^{-} cells.** Cells were imaged by DIC microscopy (Eclipse TE2000-E) while chemotaxing under agarose toward folate. Frames were acquired every 2 s, and the frame rate is 10 frames/s.

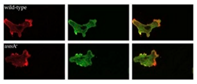

Video 2. **Actomyosin dynamics in chemotaxing wild-type and *was*A^{-} cells.** Cells expressing LifeAct-mRFP (red) and GFP-MHC II (green) were imaged by spinning disc microscopy (Revolution XD) while chemotaxing under agarose toward folate. Frames were acquired every 2 s, and the frame rate is 10 frames/s.

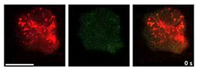

Video 3. **WASP recruitment at the sites of clathrin pit internalization.** Cells were transfected with GFP-WASP and RFP clathrin heavy chain, and imaged using a custom-built TIRF microscope. A green GFP-WASP spot is visible appearing as most of the red clathrin spots disappear from the TIRF field. Frames were acquired every 2 s, and the frame rate is 10 frames/s.

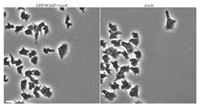

Video 4. **Exaggerated tails at the rears of *was*A^{-} cells.** Cells were imaged by low magnification phase-contrast microscopy (Eclipse TE2000-E) while chemotaxing under agarose toward folate. Frames were acquired every 15 s, and the frame rate is 15 frames/s.

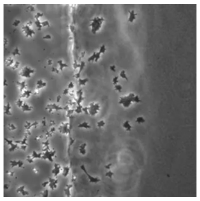

Video 5. **Lack of movement in *scar/wasp* double mutants.** *scar/wasp* knockout cells were imaged migrating under agarose using phase-contrast microscopy. Frames were acquired every 15 s, and the frame rate is 15 frames/s.

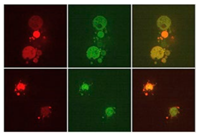

Video 6. **Actin in *scar/wasp* double mutants is dynamic.** SIKO/*wasp*$^{-}$ cells were transfected with GFP-ArpC4 and RFP-LifeAct, grown with (top) and without (bottom) DOX, and incubated with 2 µM latrunculin A. The dox^{+} cells are phenotypically *scar*$^{+}$/*wasp*$^{-}$, and dox^{-} are *scar*$^{-}$/*wasp*$^{-}$. At the start of the video, the latrunculin A was washed away and replaced with fresh medium. Both sets of cells recover F-actin with approximately equal kinetics, but Arp2/3 localizes only when SCAR is present.

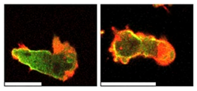

Video 7. **Myosin II localization in triple mutants.** Cells were transfected with GFP-myosin II heavy chain and RFP-LifeAct and observed while migrating. Left: *scar*$^{-}$/*ddia2*$^{-}$ cell. Right: *scar*$^{-}$/*wasp*$^{-}$/ *ddia2*$^{-}$ cell. Frames were acquired every 5 s, and the frame rate is 10 frames/s.
